# High-affinity optimization potential of the virus neutralizing antibody with twin cysteine-stabilized complementarity-determining region 3

**DOI:** 10.3389/fcimb.2025.1693895

**Published:** 2026-01-14

**Authors:** Jing Li, Dan-Dan Zeng, Qi Yin, Sen Zhang, Dong-Sheng Zhao, Yue Zhang, Zhang Zhang, Fan Tong, Zhong-Peng Zhao, Tao Jiang, Guang-Yu Zhao, Gang Dong

**Affiliations:** 1 State Key Laboratory of Pathogen and Biosecurity, Academy of Military Medical Sciences, Beijing, China; 2 College of Veterinary Medicine, Shanxi Agricultural University, Taigu, Jinzhong, China; 3 Academy of Military Medical Sciences, Beijing, China; 4 Laboratory of Advanced Biotechnology, Academy of Military Medical Sciences, Beijing, China

**Keywords:** artificial intelligence, complementarity-determining region 3, neutralizing antibody, SARS-CoV-2, twin-cysteine motif

## Abstract

**Introduction:**

The optimization of neutralizing monoclonal antibodies (NMAbs) is crucial to counter viral evolution. The structural stability of the heavy-chain complementarity-determining region 3 (H3 CDR) significantly influences affinity maturation potential, yet its impact on computational optimization remains unclear.

**Methods:**

This study employed an artificial intelligence (AI) model to optimize two categories of SARS-CoV-2 NMAbs: one featuring a conformationally stabilized H3 CDR via a twin cysteine motif, and another with flexible H3 CDR loops. Optimized antibody derivatives were evaluated for binding affinity to the SARS-CoV-2 spike protein, pseudovirus and live virus neutralization, and in vivo efficacy in a murine infection model. Structural analyses were conducted to elucidate interaction mechanisms with the angiotensin-converting enzyme 2 (ACE2) receptor.

**Results:**

H3 CDR stabilization via twin cysteines markedly enhanced AI-driven optimization efficacy. Optimized derivatives from the stabilized antibody category exhibited improved binding affinity and superior neutralization potency against both pseudotyped and authentic SARS-CoV-2 viruses. Structural analyses revealed optimized antibodies formed tighter interactions with the ACE2 receptor, including enhanced binding between key residues and ACE2, which correlated with biological efficacy. In contrast, antibodies lacking H3 CDR stabilization showed no affinity improvement after the same optimization process. In vivo, optimized antibodies effectively suppressed viral replication and reduced viral loads in infected mice. Mechanistically, the twin cysteine stabilization minimized structural perturbations caused by affinity-enhancing mutations, unlocking the optimization potential of the H3 CDR.

**Discussion:**

These findings establish that conformational stabilization of the H3 CDR in seed antibodies is a critical determinant for successful AI-driven affinity maturation. The study proposes a strategic framework for antibody development that prioritizes structurally stabilized H3 CDR regions, offering a robust approach to generating high-potency therapeutics against rapidly evolving viral pathogens.

## Highlights

- Stabilization of H3 CDR potentializes the affinity optimization of neutralizing antibody against SARS-CoV-2 virus.
- Two optimized AZD8895 NMAbs neutralize SARS-CoV-2 viruses more efficiently.
- The optimized SARS-CoV-2 NMAb AZD8895–25 indicates an incremental interaction with SARS-CoV-2 Spike.

## Introduction

The wide use of therapeutic agents such as chemical inhibitors (Lei et al., 2022) and neutralizing monoclonal antibodies (NMAbs) (Ambrose et al., 2023; Siripongboonsitti et al., 2023) has exerted a dominant role against the infection with severe acute respiratory syndrome 2 (SARS-CoV-2) (Zhou et al., 2020), during the Coronavirus Disease 2019 (COVID-19) pandemic (Hao et al., 2021). In particular, SARS-CoV-2 NMAbs directly target the virus infection and thus alleviate outcomes of either COVID-19 outpatients (Gottlieb et al., 2021; Weinreich et al., 2021) or hospitalized patients (Bender et al., 2022; Suryadevara et al., 2021). Screening the receptor-binding domain (RBD)-specific monoclonal Abs from convalescent individuals with COVID-19 has been the dominant strategy for developing Anti-SARS-CoV-2 NMAbs, from their antigen-specific B cells in the peripheral blood mononuclear cells (Brouwer et al., 2020; Ju et al., 2020). They can also be generated by screening naive or synthetic phage-displayed human antibody libraries (Parray et al., 2020; Zost et al., 2020). However, high mutation-caused immune escape has posed challenge to the effectiveness of the NMAbs (Cao et al., 2022; VanBlargan et al., 2022) against SARS-CoV-2. Thus, timely affinity optimization is vital to maintain their effectiveness, in response to SARS-CoV-2 variation. Experimental screening for key residues in the antibody complementarity-determining region (CDR) with deep mutational scanning (DMS) has facilitated NMAb optimization (Dong et al., 2021; Greaney et al., 2022). Taken together, these biological screening strategies were time-consuming and could not catch up with the virus variation.

Artificial intelligence (AI) methods have dramatically facilitated COVID-19 research and control efforts (Li et al., 2022; Nan et al., 2022) as well as NMAbs optimization (Chen and Wei, 2022; Luo et al., 2023; Zhao et al., 2023). The application of machine learning (ML) models is gaining prominence in optimizing antibody affinity. ML can discriminate against deleterious *vs*. non-deleterious mutations in the CDR of Anti-SARS-CoV-2 NMAbs (Clark et al., 2023). Embedding of antibody sequences with language model (Luo et al., 2023) and language model-based generative adversarial network improved the antibody optimization ability for Anti-SARS-CoV-2 NMAbs (He et al., 2024; Luo et al., 2023; Zhao et al., 2023). The language model embedding was also outperformed in representing the SARS-CoV-2 receptor binding protein of Spike (Ito et al., 2025; Liu et al., 2025). The main challenge for antibody development is the sequence design and structure prediction of the Heavy (H) CDR3 loop. The H3 loop matured in affinity via undergoing a mechanism of naturally *in vivo* somatic recombination (Rees, 2020), from a human antibody repertoire of ~10^15^ in size (Briney et al., 2019). Such a huge sample number and even a much higher theoretical number of ~10^18^ (Rees, 2020) challenge the AI-facilitated design and optimization of NMAbs, causing much more difficulty for accurately predicting and modeling the H3 CDR loop. Thus, the primary task for AI optimization of virus NMAbs was the seed antibody selection, based on its optimization potential. Such optimization potential was defined as the sequence- and structure-based potential of an antibody to achieve incremental properties, such as affinity, post-optimization.

The present study was designed to explore the influence of H3 CDR stabilization on the affinity optimization potential of a virus-neutralizing antibody. Two groups of SARS-CoV-2 NMAbs with or without cysteine-stabilized H3 CDR were compared for their *in vitro* and *in vivo* virus neutralizing activity and Ab–Ag interaction, post-AI optimization. Our results highlight the high-affinity optimization potential of the NMAbs with cysteine-stabilized H3 CDR.

## Materials and methods

### Antibody optimization based on seed antibodies

Five commercial and sequence-available monoclonal antibodies (AZD8895, Sotrovimab, REGN10933, REGN10987, and MW05) targeting the RBD of the SARS-CoV-2 were successfully expressed *in vitro* and selected as seed antibodies, with another antibody named LY1404 failed to *in vitro* express. These antibodies were optimized using the previously developed Language Model Guided Antibody Generative Adversarial Network (AbGAN-LMG) with the BERT2Dab language model (Zhao et al., 2023) to represent antibody sequences (**Supplementary Table 1**). Based on a comprehensive evaluation criterion of “affinity as the primary factor, supplemented by other properties and expert judgment”, 5 candidate antibodies were chosen for each seed antibody, resulting in a total of 30 antibodies selected for subsequent protein expression and experimental validation.

### Construction, expression, and purification of NMAbs

The heavy-chain and light-chain plasmids of the constructed 30 antibodies were transferred to the JM108 cloning strain. Transfection-grade plasmids were extracted using a plasmid extraction kit (QIAGEN, 12945). These plasmids were then transfected into HEK293 mammalian cells for transient expression with the aid of Lipofectamine 3000 (Invitrogen, L3000075). The resulting proteins were purified using the HiTrap Protein A HP (Cytiva, 17040301).

The expression level of mAbs was examined with SDS-PAGE. The antibody sample was mixed with a loading buffer and heated in boiling water for 10 min to ensure complete denaturation of the proteins. After centrifugation, the sample was cooled to room temperature before loading. Antibody samples and marker proteins were loaded into the gel wells. Following electrophoresis, the gel was stained with a Coomassie Blue staining solution (Solarbio, C8430), which was then discarded. The gel was washed with double-distilled water (ddH_2_O) until the background was clear, revealing distinct protein bands. After SDS-PAGE, Western blot (WB) was performed to specifically examine the expression level and the purification efficacy of NMAbs. The mAb proteins were transferred to a polyvinylidene fluoride (PVDF) membrane (Merck Millipore, IPVH00010), which was subsequently sealed with skim milk. The membrane was then treated directly with a horseradish peroxidase (HRP)-conjugated anti-human Fc antibody. Finally, the membranes were rinsed with tris-buffered saline containing Tween (TBST), and an imaging system was used to obtain images.

### Bio-layer interferometry and surface plasmon resonance

Bio-layer interferometry (BLI) analysis was performed on a Gator™ label-free bioanalysis system. Firstly, the test antibody was diluted to 5 μg/mL using a Q-Buffer, and immobilized onto Anti-HIgG Fc probes biosensors. Secondly, the SARS-CoV-2 spike RBD protein (Sino Biological, 40592-V08H) was prepared as twofold serial dilutions (20, 10, 5, 2.5, 1.25, 0.625, and 0.3125 μg/mL) with Q-Buffer used as the blank. The working procedure included baseline 1 for 120 s, loading for 300 s with the threshold limited to 1 nm, baseline 2 for 120 s, association for 300 s, and dissociation for 600 s. Kinetic values were calculated using the Gator data analysis software with a 1:1 binding model. Finally, the raw data were collected and the GraphPad Prism 9.0 software was used for drawing.

The affinity of antibodies binding to the SARS-CoV-2 RBD was detected using a BiacoreS200 instrument (GE Healthcare, Little Chalfont, UK). Briefly, recombinant Fc-fused SARS-CoV-2 RBD protein (5 μg/mL) was captured on a Sensor Chip Protein A (GE Healthcare). Recombinant His_6_-tagged mAb at various concentrations was flowed over the chip surface in 10 mM HEPES (pH 7.4), 150 mM NaCl, 3 mM EDTA, and 0.05% surfactant P20 buffer. The sensorgram was analyzed using the Biacore S200 software, and the data were fitted to a 1:1 binding model.

**Figure 1:**
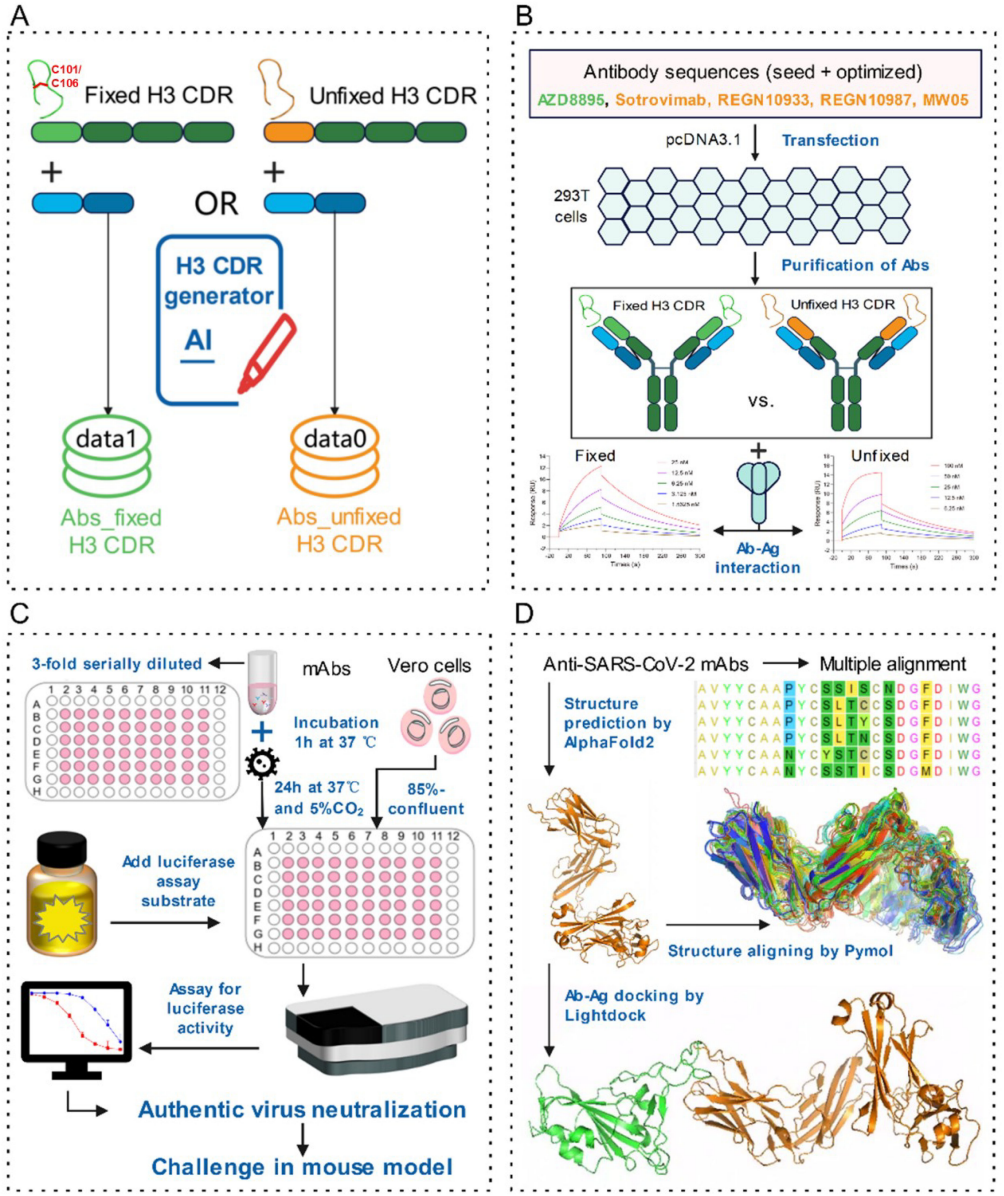
The process flow diagram for antibody optimization, expression, and functional verification. **(A)** Optimization of seed antibodies with different H3 CDR structures using the AbGAN-LMG artificial intelligence model. **(B)** Protein expression, purification, and affinity characterization of five sets of seed antibodies and their optimized variants. **(C)** Validation of viral neutralization capacity for AZD8895 and its optimized counterparts. **(D)** Structure prediction and alignment of anti-SARS-CoV-2 NMAbs. Abs_fixed H3 CDR was characterized with a twin cysteine (101C and 106C) in H3 CDR, whereas there was no twin cysteine in the H3 CDR of Abs_unfixed H3 CDR.

**Figure 2:**
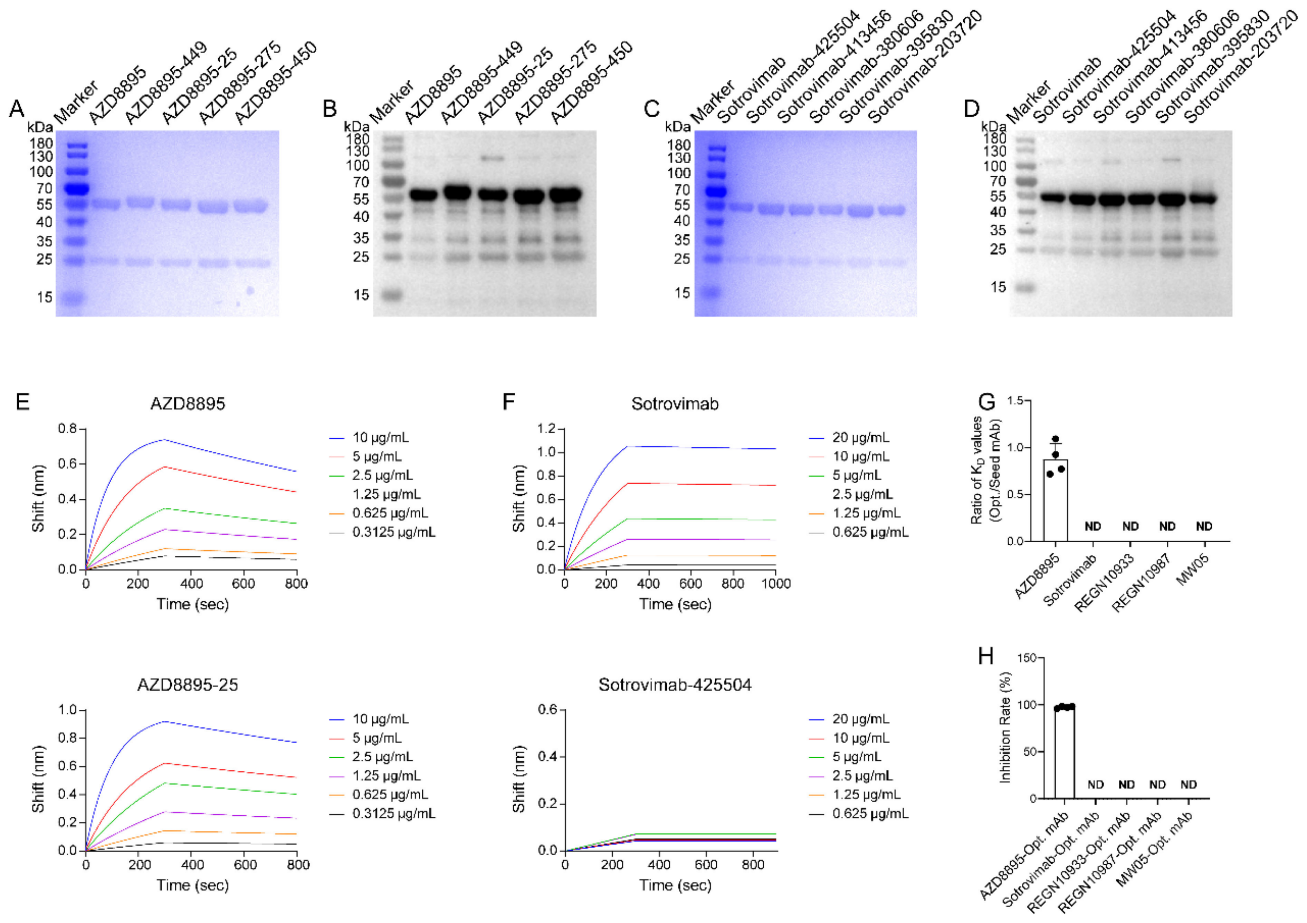
Expression of NMAbs and functional validation. **(A)** SDS-PAGE analyses of the seed antibody AZD8895 and four other optimized NMAbs (AZD8895-25, AZD8895-275, AZD8895-449, and AZD8895-450). The molecular weight marker (in kDa) is indicated on the left. **(B)** Western blot analyses of the seed antibody AZD8895 and four other optimized mAbs. The molecular weight marker (in kDa) is indicated on the right. **(C)** SDS-PAGE analyses of the seed antibody Sotrovimab and five other optimized NMAbs (Sotrovimab-425504, Sotrovimab-413456, Sotrovimab-380606, Sotrovimab-395830, and Sotrovimab-203720). The molecular weight marker (in kDa) is indicated on the left. **(D)** Western blot analyses of the seed antibody Sotrovimab and five other optimized NMAbs. The molecular weight marker (in kDa) is indicated on the right. **(E)** Binding kinetics between AZD8895 and its optimized antibody AZD8895–25 with the SARS-CoV-2 spike protein were measured using BLI. **(F)** Binding kinetics between Sotrovimab and its optimized antibody Sotrovimab-425504 with the SARS-CoV-2 spike protein were measured using BLI. **(G)** The ratio of *K*_D_ values between the optimized NMAbs and the seed antibody. For AZD8895 and its optimized mAbs, calculation of the mean ratio for the four optimized candidates yielded a value of 0.88, indicating a superior affinity of the optimized antibodies compared to the parental seed antibody. For optimized antibodies derived from Sotrovimab, REGN10933, REGN10987, and MW05, ***K*_D_** values were not detectable (ND) due to lack of binding. **(H)** Inhibition rate of optimized NMAbs (derived from various seed antibodies) against the SARS-CoV-2 pseudovirus. Optimized antibodies based on the AZD8895 exhibited an average pseudovirus inhibition rate of 97.40%, while inhibition rates of other seed candidates were not detectable (ND).

The presented sensorgrams and *K*_D_ values in **Figures 1E, F** and **2A, B** and **Supplementary Table 1** are representative of at least two independent experiments performed with freshly prepared proteins.

### Enzyme-linked immunosorbent assay

The inactivated SARS-CoV-2 virus (wild type, Wuhan-Hu-1) was diluted in a coating solution (5 × 10^4^ PFU/mL) and incubated overnight at 4°C in a transparent 96-well plate. After washing with phosphate-buffered saline with Tween 20 (PBST), the wells were sealed with PBST containing 3% bovine serum albumin (BSA) at 37°C for 1.5 h. Following another wash, diluted antibody samples were added and incubated at 37°C for 2 h. A goat anti-human secondary antibody (Beyotime, A0201), diluted in PBST with 1% BSA, was added and incubated at 37°C for another 1.5 h after washing. Subsequently, TMB substrate (Beyotime, P0209) was added and incubated for 15 min in the dark. A termination solution of 1 M H_2_SO_4_ was then added to stop the reaction, and the OD_450_ value was detected using a detector. The half-maximal effective concentration (EC_50_) was calculated using GraphPad Prism 9.0 software as previously described.

### Pseudovirus neutralization assay

Antibody samples and pseudovirus (based on SARS-CoV-2 wild-type, Wuhan-Hu-1) were diluted in DMEM complete medium (Gibco, 1196511). Serially diluted antibody samples were mixed with an equal volume of diluted pseudovirus solution in a 96-well cell culture plate and incubated at 37°C in a 5% CO_2_ atmosphere for 1 h. Following this, 100 μL of Vero cells at a density of 2 × 10^5^ cells/mL was added to each well and incubated at 37°C in a 5% CO_2_ atmosphere for 24 h. After incubation, the cell culture plate was removed, and Bio-Lite luciferase assay reagent (Vazyme, DD1204) was equilibrated to room temperature. A bottle of Bio-Lite luciferase assay buffer was mixed with Bio-Lite luciferase assay substrate thoroughly. Then, the supernatant (150 μL) from each well was aspirated and discarded. Bio-Lite luciferase assay reagent was added, and the plate was agitated for 2 min to lyse the cells adequately. The liquid was then transferred to a corresponding white opaque chemiluminescence assay plate for chemiluminescence detection using a multifunctional enzyme marker equipped with chemiluminescence detection capability, allowing for measurement of relative light unit (RLU) values. The pseudovirus infection inhibition rate was calculated as previously described.

### *In vitro* neutralization assay against SARS-CoV-2 virus

The live SARS-CoV-2 neutralization assay was performed as previously described (Bewley et al., 2021). Briefly, SARS-CoV-2 (Omicron BA.5) at an amount equivalent to 100 median tissue culture infective doses (TCID_50_) was incubated with NMAbs at varying concentrations for 1 h at 37°C. The mAb–virus mixture was then incubated with Vero cells for 72 h at 37°C in the presence of 5% CO_2_. The cytopathic effect (CPE) was observed daily, and the neutralizing activity of the antibody was reported as the 50% neutralization dose (ND_50_). The Reed–Muench method was used to calculate the ND_50_ value for each mAb.

### Structure prediction of MNAbs and SARS-CoV-2 receptor and structure docking between NMAb and SARS-CoV-2 receptor

The three-dimensional (3D) structures of NMAbs and the SARS-CoV-2 receptor angiotensin-converting enzyme 2 (ACE2) were predicted by Alphafold2 as previously reported (Jumper et al., 2021; Zhang et al., 2024). In brief, the prediction was performed under a created virtual environment of alphafold, successively with commands of “conda activate alphafold” and “python/home/inspur/git_package/alphafold-main/docker/run_docker.py --fasta paths =/home/inspur/git_package/alphafold-main/AZD8895.fasta (for AZD8895 as an example) --max_template_data = 2020-05–14”. The predicted AZD8895.pdb or ACE2.pdb was visualized with PyMOL (version 4.60) via terminal under an ubuntu system. To explore a possible interaction between NMAb and ACE2, a protein–protein docking using residue restraints was performed with lightdock under reported protocols (Jimenez-Garcia et al., 2018; Roel-Touris et al., 2020). In brief, a setup step was run, enabling flexibility (-anm), excluding the terminal oxygens (--noxt) and all hydrogens (--noh) and water (--now), and specifying the residue restraints that will bias the docking simulation. A total of 200 swarms were simulated, redundant predictions intra-swarm were clustered, and the predictions compatible with the provided restraints were filtered out. The 3D structures of NMAb-ACE2 docking file were manually checked with the guidance of rank_by_rmsd.list, rank_by_luciferin.list, and rank_by_scoring.list. The free energy of the wild-type AZD8895 and a simulated mutant type of AZD8895 with simulated mutation of C101A and C106A (with the C101/C106 disulfide bond broken) was calculated with the ddG package of pyrosetta (https://github.com/Jamalijama/TwinCysteineCDRH3).

### Therapeutic efficacy evaluation of the antibodies

Female K18-hACE2 transgenic mice (GemPharmatech Co. Ltd., China), aged 6–8 weeks, were housed in a biosafety level 3 facility with *ad libitum* access to standard pellet feed and water. All animal experiment protocols were reviewed and approved by the Institutional Animal Care and Use Committee of the Academy of Military Medical Sciences (Permit number: IACUC-IME-2022-045). Mice (*n* = 3–5 per group) were intranasally inoculated with 2 × 10^3^ PFU of the SARS-CoV-2 after intraperitoneal anesthetization with sodium pentobarbital. One day post-virus challenge, the mice were intraperitoneally injected with antibody AZD8895–275 or AZD8895 at a dose of 10 mg/kg according to previously published studies (Jimenez-Garcia et al., 2018; Roel-Touris et al., 2020). The mice in the mock group received an equal volume of PBS. The body weights were recorded daily for 3 days post-infection. Additionally, mouse lung tissues were collected 3 days post-infection for viral load assessment using real-time RT-PCR. Briefly, the total RNA from the lung tissues that were lyzed using TRIzol reagent was extracted and reverse-transcribed to cDNA using a reverse transcription kit (Takara, RR037Q). Subsequently, viral copies targeting the E gene of SARS-CoV-2 were quantified by real-time PCR using the following probe and primers: E_Sarbeco_F: ACAGGTACGTTAATAGTTAATAGCGT; E_Sarbeco_P1: FAM-ACACTAGCCATCCTTACTGCGCTTCG-BBQ; E_Sarbeco_R: ATATTGCAGCAGTACGCACACA.

### Statistical analysis

All statistical analyses were performed using GraphPad Prism 9.0 (GraphPad Software, San Diego, CA, USA). For comparisons between two groups with normally distributed data, unpaired Student’s *t*-test was applied. Multi-group analyses were conducted using one-way analysis of variance (ANOVA). Significance levels in figures are denoted as follows: ns (not significant, *p* ≥ 0.05), *p < 0.05, **p < 0.01, and ***p < 0.001.

## Results

### Pipeline to evaluate affinity optimization potential of SARS-CoV-2 NMAbs

We initially optimized two categories of seed NMAbs using the previously established AbGAN-LMG AI model (**Figure 1A**). One category featured a disulfide bond fixation within the H3 CDR loop, exemplified by antibody AZD8895, while the other category comprised antibodies with relatively flexible H3 CDR loops, specifically Sotrovimab, REGN10933, REGN10987, and MW05. Based on these five seed NMAbs, sequences for five sets of optimized antibodies were generated. From each set, the top five sequences were selected after scoring and screening. After that, these five sets of NMAbs (one seed antibody and its corresponding optimized antibodies) were subjected to expression and purification processes (**Figure 1B**). Their functional attributes were subsequently validated through an array of wet-lab experiments (**Figure 1C**). Furthermore, the influence of H3 CDR flexibility on affinity enhancement during the optimization process was further scrutinized via molecular docking analyses (**Figure 1D**).

### High optimization potential of the seed SARS-CoV-2 NMAb with stabilized H3 CDR

**Figure 2A** shows the SDS-PAGE results for the seed antibody AZD8895 (with a fixed H3 CDR) and its optimized variants (AZD8895-25, AZD8895-275, AZD8895-449, and AZD8895-450), indicating successful expression of four out of the five optimized antibodies. Under reducing conditions, these NMAbs dissociated into heavy and light chains of approximately 60 and 25 kDa, respectively, consistent with the expected antibody sizes. Target bands were also detected at the corresponding positions by WB (**Figure 2B**). **Figures 2C, D**, along with **Supplementary Figure S1**, present the expression profiles of four seed antibodies with a fixed H3 CDR structure (Sotrovimab, REGN10933, REGN10987, and MW05) and their optimized variants. The results indicate that out of 20 optimized antibodies, 17 were successfully expressed (5 for Sotrovimab, 4 for REGN10933, 5 for REGN10987, and 3 for MW05), with bands observed at their theoretical molecular weights. The purified antibodies showed comparable expression as assessed by the quantitative densitometric analysis of the WB bands (**Supplementary Figure S1**), confirming that the expression levels across all variants were not significantly different. The BLI method was then utilized to evaluate the binding affinity of five sets of antibodies to the SARS-CoV-2 spike protein (**Figures 2E, F**; **Supplementary Figure S2**; **Supplementary Table 1**). It was observed that, aside from the optimized antibody based on AZD8895, which demonstrated binding capability to the spike protein, the optimized antibodies derived from non-stabilized seed antibodies (Sotrovimab, REGN10933, REGN10987, and MW05) showed no detectable binding to the SARS-CoV-2 spike protein in BLI assays. **Figure 2G** illustrates the ratio of dissociation constants (*K*_D_) between the optimized and seed antibodies, indicating that the optimized antibody of AZD8895 possesses a *K*_D_ value comparable to or even superior to that of the seed antibody (with an average *K*_D_ value of 0.88), while the optimized antibodies based on the other four seed NMAbs could not detect any *K*_D_ values. Preliminary functional validation of the antibodies was conducted through pseudovirus neutralization assays against SARS-CoV-2. Notably, the series of antibodies optimized from AZD8895 demonstrated strong pseudovirus neutralization capabilities, while the series derived from the other four seed antibodies did not exhibit ideal neutralization efficacy (**Figure 2H**). These results collectively demonstrate that the twin-cysteine stabilized H3 CDR is a critical determinant for successful AI-driven affinity optimization, as only antibodies derived from the stabilized seed (AZD8895) retained binding and neutralization function.

### Affinity and neutralization increment of the H3 CDR-stabilized SARS-CoV-2 NMAb post-optimization

**Figure 3:**
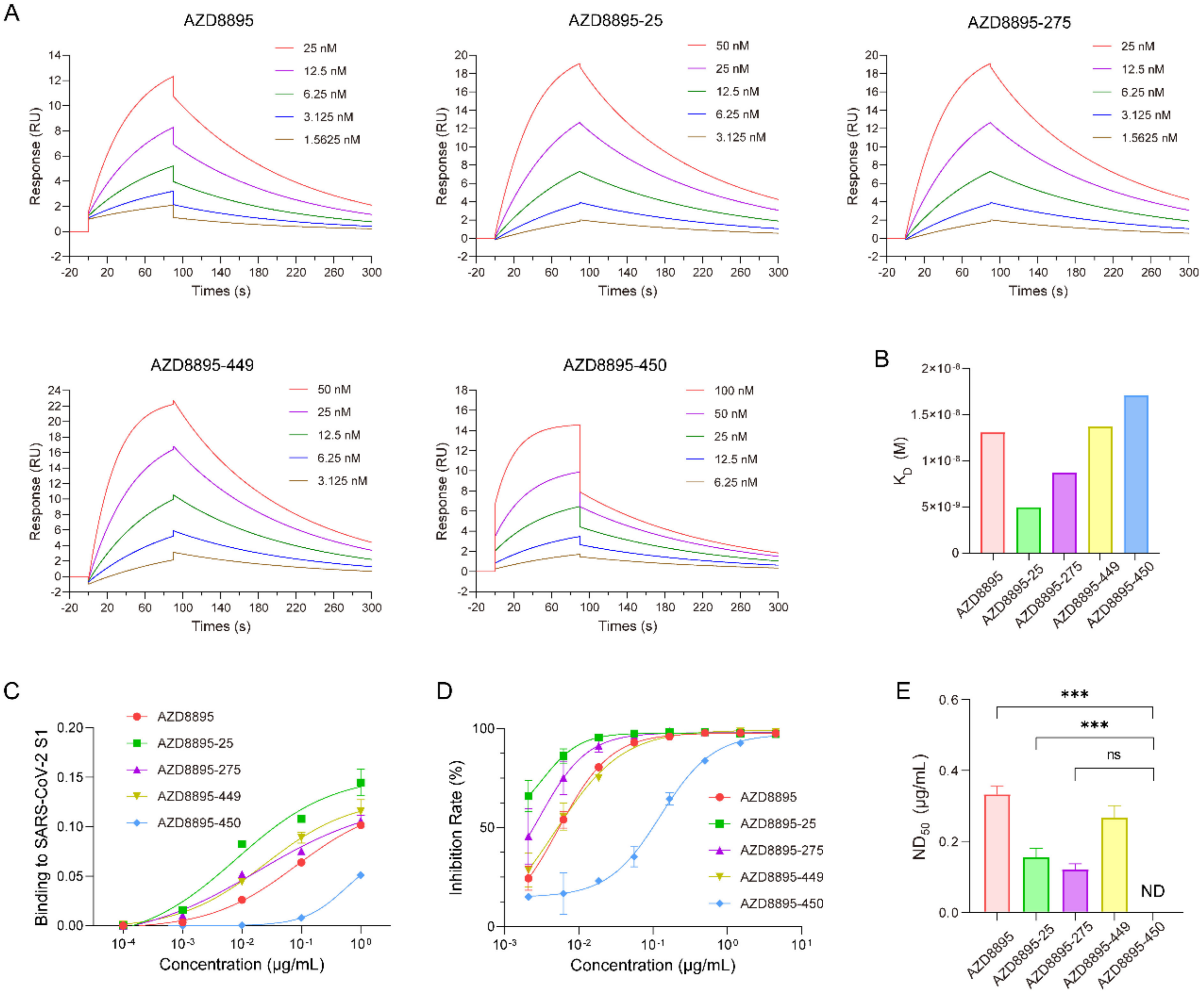
Enhanced antigen-binding capacity, spike-specific targeting, and SARS-CoV-2 neutralization by optimized NMAbs. **(A)** SPR analysis of binding between AZD8895 and its optimized NMAbs with SARS-CoV-2 S1 protein. **(B)***K*_D_ values for five NMAbs. Specific *K*_D_ values: AZD8895, 12.6 nM; AZD8895-25, 4.79 nM; AZD8895-275, 8.42 nM; AZD8895-449, 13.2 nM; and AZD8895-450, 16.5 nM. **(C)** Detection of binding affinity between five NMAbs and inactivated SARS-CoV-2 virus by ELISA. Data are shown as mean ± SD of technical triplicates from one representative experiment. The experiment was repeated twice with consistent results. **(D)** Pseudovirus neutralization curves of five antibodies. Data are presented as mean ± SD from three independent experiments. **(E)** Neutralizing activity of five antibodies against the authentic SARS-CoV-2. Neutralizing activity of mAb was recorded as the concentration of mAb in complete inhibition of SARS-CoV-2-induced CPE in at least 50% of the wells (ND_50_). The data are expressed as mean ND_50_ ± the SD (*n* = 3). Specific ND_50_ values: AZD8895, 0.34 ± 0.02 μg/mL; AZD8895-25, 0.16 ± 0.03 μg/mL; AZD8895-275, 0.12 ± 0.02 μg/mL; AZD8895-449, 0.27 ± 0.03 μg/mL; and AZD8895-450, not detected. The statistical comparison was performed using an unpaired Student’s *t*-test. ***p < 0.001; ns, not significant.

In light of the strong performance of AZD8895 and its optimized antibodies, further validation of their biological functions was conducted. Given that the primary focus during the optimization of antibodies was to enhance affinity, surface plasmon resonance (SPR) was utilized to measure the binding affinity of these antibodies to the SARS-CoV-2 spike protein. The results showed that the *K*_D_ value of the seed antibody AZD8895 was 12.6 nM, while the *K*_D_ of AZD8895–25 and AZD8895–275 were 4.79 and 8.42 nM, respectively, indicating a significant improvement in affinity. Antibodies AZD8895–449 and AZD8895–450 did not show a noticeable enhancement in affinity (**Figures 3A, B**). To characterize their functionality, the interaction between these antibodies and SARS-CoV-2 was investigated. An enzyme-linked immunosorbent assay (ELISA) was performed to assess the binding of the NMAbs to inactivated SARS-CoV-2. As shown in **Figure 3C**, three modified antibodies—AZD8895-25, AZD8895-275, and AZD8895-449—demonstrated strong, dose-dependent reactivity with inactivated virus, similar to the seed antibody AZD8895. Notably, the modified antibodies exhibited significantly enhanced binding affinity compared to the seed antibody: the EC_50_ for AZD8895 was 0.083 μg/mL, while AZD8895-25, AZD8895-275, and AZD8895–449 achieved EC_50_ values of 0.008, 0.019, and 0.025 μg/mL, respectively. In contrast, antibody AZD8895–450 did not exhibit significant binding under the tested conditions. Next, the neutralizing activity of these NMAbs against SARS-CoV-2 pseudovirus was evaluated in Vero cells (**Figure 3D**). Antibody AZD8895-449 (IC_50_ = 4.8 ng/mL) showed comparable neutralization capabilities to the seed antibody AZD8895 (IC_50_ = 5.3 ng/mL). Antibodies AZD8895–25 and AZD8895–275 exhibited significantly enhanced neutralization efficacy, achieving IC_50_ values of 3.2 and 3.0 ng/mL, respectively. The live virus neutralization ability was further assessed (**Figure 3E**), revealing that the neutralization of antibody AZD8895–449 was not significantly different from the seed mAb. However, the antibodies AZD8895–25 and AZD8895–275 displayed enhanced neutralization effects compared to AZD8895, while AZD8895–450 did not neutralize the live virus. The IC_50_ and ND_50_ values are listed in **Supplementary Figure S3**, along with a correlation analysis. The IC_50_ value for AZD8895–449 was similar to that of AZD8895, whereas the IC_50_ values for AZD8895–25 and AZD8895-275 (2.63 and 3.0 ng/mL, respectively) were notably lower, demonstrating superior neutralization efficacy. Similarly, the ND_50_ values for AZD8895-25 (0.16 μg/mL) and AZD8895-275 (0.12 μg/mL) indicated improved neutralization effects against live SARS-CoV-2 compared to seed antibody. The strong correlation (*R*² = 0.9108) between pseudovirus (IC_50_) and live virus (ND_50_) neutralization efficacy validates the reliability of the pseudovirus assay as a predictive tool for authentic virus neutralization. However, the correlation is not perfect. The slight deviations observed could be attributed to the more complex nature of the live virus infection cycle than the pseudovirus assay system, and potentially also to the genetic divergence between the Wuhan-Hu-1 and Omicron BA.5 Spike proteins. The results of correlation analysis showed that, except for antibody AZD8895-450, which lacks actual virus neutralization capability, the IC_50_ and ND_50_ values of the remaining four antibodies show significant correlation (*R*² = 0.9108, *p* < 0.05). The significant enhancement in affinity (*K*_D_) and neutralization efficacy (IC_50/_ND_50_) of optimized antibodies like AZD8895–25 and AZD8895-275, but not AZD8895-450, validates the functional success of the optimization and suggests a structure–function relationship dependent on specific mutations.

### Binding increment in the 3D structure of the H3 CDR-stabilized SARS-CoV-2 NMAb post-optimization

**Figure 4:**
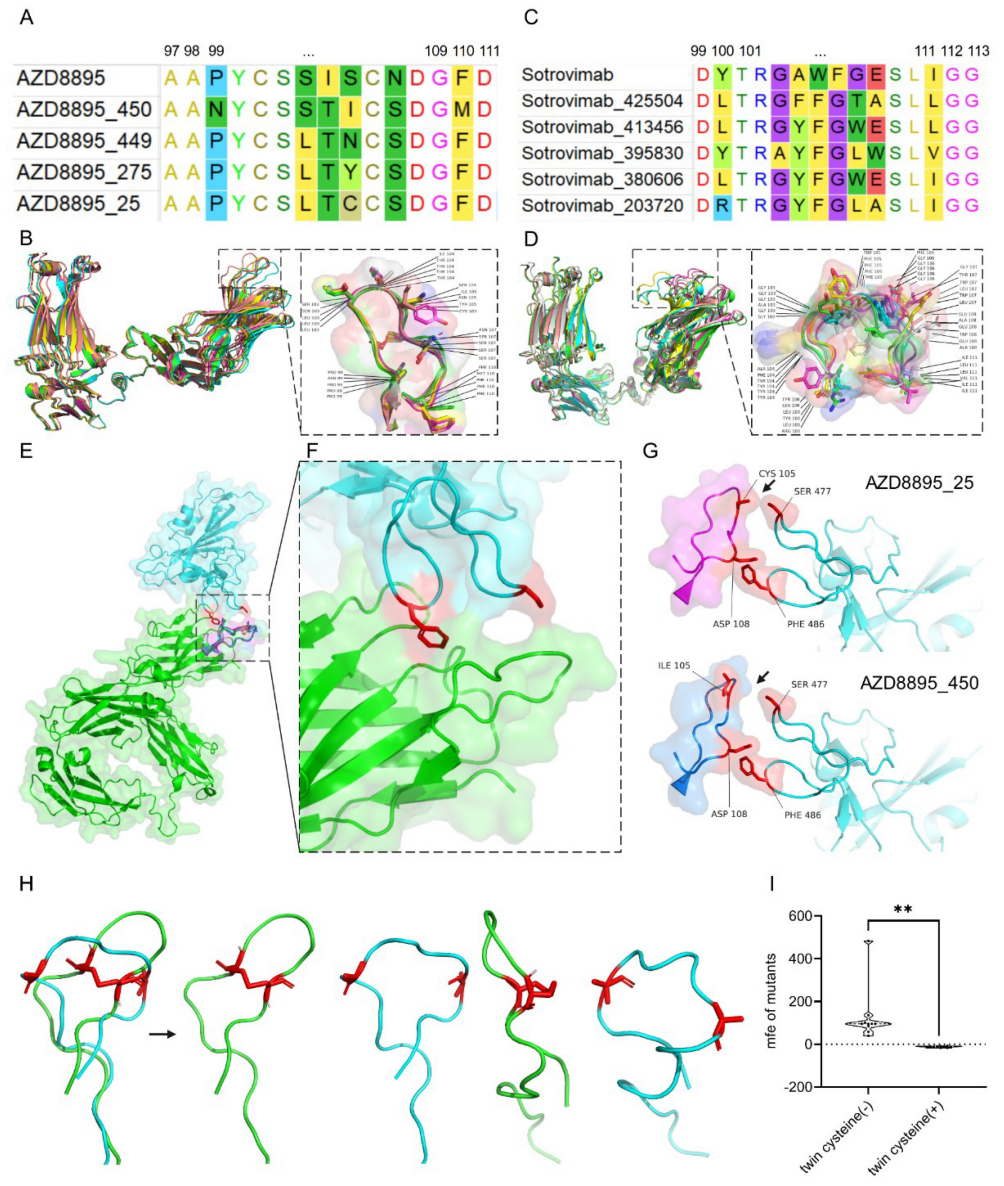
Sequence alignment and structure docking of AZD8895-based optimized antibodies. Sequence alignment **(A)** and 3D structure alignment **(B)** of AZD8895 and its optimized antibodies, which were stabilized with twin cysteines in H3 CDR; Alignment in sequence **(C)** and 3D structure **(D)** of Sotrovimab and its optimized antibodies. 3D structure of AZD8895 and Spike **(E)** and the zoomed interface between the two proteins **(F)**, respectively colored in green and cyan, with the predicted interacting residues in red. Mutational effect of the residues within the two cysteine-stabilized H3 CDR on the AZD8895–Spike interface **(G)** The structure of CDR H4 (residues 97 to 116) from wild type of AZD8895 (green) and of the CDR H4 (residues 97 to 116) with the mutations of C101A and C106A from AZD8895 (cyan) was predicted with Alphafold2 and visualized with PyMOL **(H**, with 101/106 residues in red). **(I)** The free energy of optimized Abs (with each amino mutation for the four optimized Ab) respectively based on the two seed Abs (C101A/C106A mutant seed *vs*. wild-type AZD8895) was calculated by pyrosetta. (**p < 0.01).

To explore a possible interaction shift between the SARS-CoV-2 receptor of ACE2 and the optimized NMAb, both proteins were predicted with Alphafold2 for their 3D structure and were docked with LightDock. Because of the alignment of the H3 CDR-stabilized SARS-CoV-2 NMAb, AZD8895, only three to four residues were mutated for the four optimized NMAbs within or near the twin cysteines (**Figure 4A**). Such residue substitution in a few sites only led to slight structural deviation of the optimized NMAbs, with a root mean square deviation (RMSD) of 1.0787 ± 0.6885, compared to the seed AZD8895 (**Figure 4B**; **Supplementary Table 2**). On the other side, the H3 CDR-nonstabilized seed antibody (Sotrovimab)-based optimized NMAbs were prone to produce as many as six to seven mutated residues under the same generating model (**Figure 4C**), and higher structure shifting (**Figure 4D**), with an RMSD of 3.3413 ± 3.4368, compared to their seed NMAb (**Supplementary Table 2**). Moreover, the increment in affinity and neutralization of optimized NMAb was not dependent on the structure waving of the NMAb (**Supplementary Table 3**), also implying the independent contribution of the twin cysteines to the optimization potential of NMAb. The enlarged interface between ACE2 and NMAb AZD8895 showed two possible interacting residue pairs of the 105SER in AZD8895 and the 477SER in ACE2 and the 108ASP in AZD8895 and the 486PHE in ACE2 (**Figures 4E, F**). In particular, the AZD8895–25 interacted more closely in the 105CYS with the 477SER in ACE2 than the interaction between the 105ILE of AZD8895–450 and the 477SER in ACE2 (**Figure 4G**). Such interacting differences are matched to the biologically validated binding and neutralizing activity of the two NMAbs. These results suggest that the greater binding and neutralizing activity of AZD8895–25 benefits from its closer binding to ACE2 in structure. The stabilization role of the twin cysteine was also indicated by the fixed DCR H3, compared to the simulated mutant of CDR H4 with C101A and C106A from AZD8895 (**Figure 4H**). Such a role was also supported by the lower free energy of the group of twin-cysteine CDR H3 loops with each of the mutations in CDR3 (**Figure 4I**; p < 0.01, **Supplementary Tables 4** and **5**).

### Incremental *in vivo* virus neutralization of the H3 CDR-stabilized SARS-CoV-2 NMAb post-optimization

**Figure 5:**
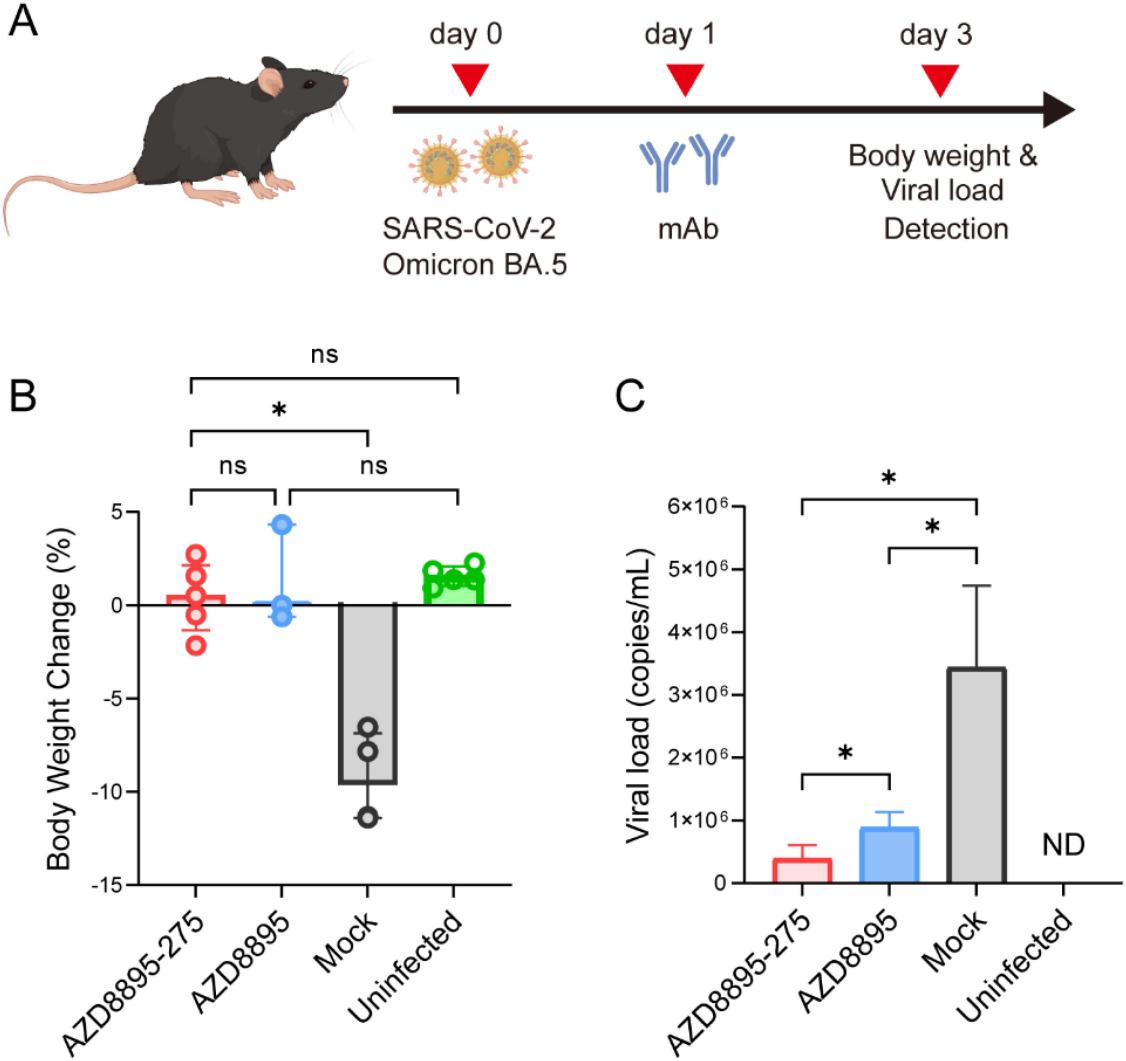
Evaluation of the therapeutic efficacy of antibodies in the K18-hACE2 transgenic mouse model of SARS-CoV-2 infection. **(A)** Therapeutic study schema. Mice were inoculated with virus on day 0, treated with antibodies on day 1, and received body weight and viral load detection on day 3. **(B)** Body weight changes in different groups of mice on day 3. *p < 0.05; ns, not significant. **(C)** Lung tissue viral load in different groups of mice on day 3. *p < 0.05. Data are presented as mean ± SD (*n* = 3–5 mice per group).

*In vivo* neutralization potential is one of the key indicators for evaluating the effectiveness of antiviral drug candidates. Our previous experiments have demonstrated that, among the optimized NMAbs, antibody AZD8895–25 exhibited the strongest virus neutralization capability. We further evaluated the therapeutic efficacy of antibody AZD8895–275 in an animal infection model. Mice were first infected with SARS-CoV-2 and then intravenously injected with AZD8895–275 or AZD8895 at a single dose of 10 mg/kg 1 day post-infection. Mice in the mock group received an equal volume of PBS. Weight changes and viral loads were assessed at 3 dpi (**Figure 5A**). The results indicated that mice in the mock group experienced a 9.28% decrease in body weight, whereas mice treated with AZD8895–275 or AZD8895 maintained stable weights, with no significant differences in weight changes between the two antibody-treated groups. This suggests that AZD8895–275 did not confer a significant advantage over AZD8895 in terms of weight maintenance in infected mice (**Figure 5B**). Despite the comparable prevention of weight loss by both antibodies, the significantly lower lung tissue viral load in AZD8895-275-treated mice demonstrates its superior efficacy in suppressing viral replication. A modest but statistically significant advantage in reducing viral load by AZD8895–275 against its seed antibody was indicative of genuine antiviral activity and a potential therapeutic benefit, given the rapid and high-titer viral replication in the K18-hACE2 model. Together, these *in vivo* results provide a proof-of-concept validation that the affinity-enhancing mutations identified in AZD8895–275 are functional within a living system, a finding that is strongly supported by our comprehensive *in vitro* data.

## Discussion

AI and computational technologies have substantially promoted advances in both predictive and generative tasks for biological sequences, the structure, and other properties of biomolecules. Some promising results have also been achieved by some Ab development and optimization pipelines via overcoming some limitations of traditional empirical antibody development strategies. In particular, deep learning-guided optimization of human neutralizing antibody against SARS-CoV-2 (Shan et al., 2022) and HIV (Danaila and Buiu, 2022) has been sporadically reported. However, the massive diverse space of antibody sequences (~10^15^ variants full-length antibodies) (Briney et al., 2019) challenges the selection and targeting of a seed antibody for AI optimization. In the present study, we compared the optimization potential of the neutralizing antibody against SARS-CoV-2 virus between two types of seed antibodies, either with or without H3 CDR3 stabilized by twin cysteines. Our results recognized a high optimization potential of the seed SARS-CoV-2 NMAb with twin cysteine-stabilized H3 CDR. Such potential manifested incremental Ab–Ag interaction, increased affinity, and neutralization of the optimized NMAbs from a SARS-CoV-2 NMAb with the stabilized H3 CDR by twin cysteines. It is worth mentioning that the increased affinity of the optimized SARS-CoV-2 NMAbs was confirmed at both *in vitro* and *in vivo* levels. Furthermore, the contrasting outcomes of AZD8895–25 and AZD8895–450 underscore that while the stabilized H3 CDR scaffold provides a privileged platform for optimization, conferring tolerance to mutations, the functional success remains critically dependent on the nature of the introduced substitutions. This highlights that the AI-driven approach, although powerful, does not bypass the necessity for experimental validation to sift through the generated variants.

The twin cysteine-mediated stabilization of a loop motif is vital for the structure and function of the variable regions of HIV surface proteins, which is associated with neutralization escape and breadth development (Bowder et al., 2018; Hesselman et al., 2025), the cell surface calcium-sensing receptor (Goolam et al., 2022), and the phytochelatin synthases (PCSs) (Li et al., 2020). In particular, a significant contribution has been observed by the twin cysteines to the exposure of bnAb-specific epitope in the envelope variable region 1 of HIV-1 (Hesselman et al., 2025), and to the loop stability of immunogen gp120 variable region 2 of HIV-2 (Bowder et al., 2018). On the other hand, the interdomain twin cysteine-mediated stabilization also contributes to the antibody affinity of the single-chain fragment variable (Zhao et al., 2010). Thus, we speculated that such twin cysteine-mediated fixation of a protein motif not only could prevent a dramatic structure shift caused by possible mutations in the twin cysteine-stabilized motif but also reduced its potential influence on the structure variation of its neighbor motifs. Such a hypothesis was confirmed by our results that show that the five optimized MNAbs from the twin cysteine-stabilized seed NMAb varied in 3D structure the least against the other optimized MNAbs based on no twin cysteine-stabilized seed NMAbs.

AI-based methods have made promising achievements on antibody development. Both strategies of seed antibody-based optimization (Bai et al., 2023; Kim et al., 2023) and *de novo* design (Finlay and Lugovskoy, 2019; He et al., 2024) were focused on the CDR, mainly H3 CDR of antibodies. Thus, the selection of seed H3 CDR3 was primary and vital for AI-based antibody development. Our results implied the high importance of the optimization potential of H3 CDR: a significant advantage of structure-stable H3 CDR over unstable H3 CDR was found to associate with the twin cysteine-mediated H3 CDR stabilization. Therefore, the twin cysteine-stabilized antibodies, including virus NMAbs, show great potential as seed antibody for affinity optimization and are promising candidates for *de novo* antibody design. Moreover, our results highlight the need to screen more cysteine-stabilized antibodies from the human antibody repertoire for neutralizing viruses and, thus, to develop a strategy first to screen the seed NMAbs with stabilized H3 CDR and secondly to optimize the antibody affinity via AI methods. It is important to note that this study focused on affinity optimization against only one virus target, SARS-CoV-2 wild type (Wuhan-Hu-1), and only a single variant (Omicron BA.5) was tested for the neutralizing effect of optimized NMAbs. Future work will be essential to optimize NMAbs against a panel of current and emerging SARS-CoV-2 variants and evaluate the breadth of neutralization of these optimized antibodies to assess their potential therapeutic utility. It should be noted that a non-infected control group was not included in the current study design. While the stabilization of body weight in treated groups compared to the infected mock group strongly indicates therapeutic efficacy, the inclusion of a naïve control in future studies will provide a valuable baseline for more precisely quantifying the health benefits of the treatment. Additionally, a simulated seed mutant AZD8895 with C101A and C106A substitutions indicated significantly less stabilization of the CDR3 with higher free energy and much higher free energy variation of the mutant seed-based CDR3 optimized antibodies. Of course, more experimental validation of the stabilization role of the twin cysteine would solidify the conclusion about the stabilization contribution of the twin cysteine in CDR3. The stabilization contribution of twin cysteine to H3 CDR needs to be evaluated further in other antibodies.

## Conclusion

In summary, the present study found the significant importance of twin cysteine-stabilized H3 CDR for the affinity optimization of virus NMAbs. A SARS-CoV-2 NMAb with twin cysteine-stabilized H3 CDR was successfully optimized in its *in vitro* and *in vivo* affinity to SARS-CoV-2. Our results highlight the high importance of cysteine-stabilized H3 CDR on NMAb optimization.

## Data Availability

The datasets presented in this study can be found in online repositories. The names of the repository/repositories and accession number(s) can be found in the article/**Supplementary Material**.
