# Supplementary material for: High-affinity optimization potential of the virus neutralizing antibody with twin cysteine-stabilized complementarity-determining region 3
Source: Front Cell Infect Microbiol. 2026 Jan 14;15:1693895. doi: 10.3389/fcimb.2025.1693895 (PMC12847411; doi:10.3389/fcimb.2025.1693895)
Supplement: Supplementary file 1 [file DataSheet1.pdf]

Supplementary material

Supplementary Table 1. Kon, Koff, and K_D values of seed antibodies and their optimized antibodies detected by BLI.

NMAb name	Kon ($M^{-1} s^{-1}$)	Koff (s^{-1})	K_D (M)
AZD8895	8.37×10^{4}	5.62×10^{-4}	6.71×10^{-9}
AZD8895-25	7.38×10^{4}	3.55×10^{-4}	4.81×10^{-9}
AZD8895-275	8.32×10^{4}	4.30×10^{-4}	5.17×10^{-9}
AZD8895-449	8.73×10^{4}	5.42×10^{-4}	6.20×10^{-9}
AZD8895-450	4.90×10^{4}	3.59×10^{-4}	7.33×10^{-9}
Sotrovimab	2.27×10^{4}	3.23×10^{-5}	1.42×10^{-9}
REGN10987	1.08×10^{5}	6.37×10^{-4}	5.92×10^{-9}
REGN10933	5.56×10^{4}	3.73×10^{-4}	6.70×10^{-9}
MW05	4.10×10^{4}	7.73×10^{-5}	1.89×10^{-9}

Supplementary Table 2. Heavy chain sequences of seed or optimized antibody.

NMAb name	Heavy chain sequence (N- to C-terminal)	Referance
AZD8 895	QMQLVQSGPEVKKPGTSVKVSCKASGFTFMSSAVQWVRQARGQRLEWIGWIVIGSGNTNYAQKFQERVTITRDMSTSTAYMELSSLRSEDTAVYYCAA<br>PYCSSISCNDGFDIWGQGTMVTVSSASTKGPSVFPLAPSSKSTSGGTAALGCLVKDYFPEPVTVSWNSGALTSGVHTFPAVLQSSGLYSLSSVVTVPSSSLG<br>TQTYICNVNHKPSNTKVDKRVEPKSCDKTHTCPPCPAPEFEGGPSVFLFPPKPKDTLYITREPEVTCVVVDVSHEDPEVKFNWYVDGVEVHNAKTKPREE<br>QYNSTYRVVSVLTVLHQDWLNGKEYKCKVSNKALPASIEKTISKAKGQPREPQVYTLPPSREEMTKNQVSLTCLVKGFYPSDIAVEWESNGQPENNYKT<br>TPPVLDSDGSFFLYSKLTVDKSRWQQGNVFSCSVMHEALHNHYTQKSLSLSPGK	10.1038/s41 564-021-00972-2
AZD8 895-25	QMQLVQSGPEVKKPGTSVKVSCKASGFTFMSSAVQWVRQARGQRLEWIGWIVIGSGNTNYAQKFQERVTITRDMSTSTAYMELSSLRSEDTAVYYCAA<br>PYCSLTCCSDGFDIWGQGTMVTVSSASTKGPSVFPLAPSSKSTSGGTAALGCLVKDYFPEPVTVSWNSGALTSGVHTFPAVLQSSGLYSLSSVVTVPSSSL<br>GTQTYICNVNHKPSNTKVDKRVEPKSCDKTHTCPPCPAPEFEGGPSVFLFPPKPKDTLYITREPEVTCVVVDVSHEDPEVKFNWYVDGVEVHNAKTKPRE<br>EQYNSTYRVVSVLTVLHQDWLNGKEYKCKVSNKALPASIEKTISKAKGQPREPQVYTLPPSREEMTKNQVSLTCLVKGFYPSDIAVEWESNGQPENNYK<br>TTPPVLDSDGSFFLYSKLTVDKSRWQQGNVFSCSVMHEALHNHYTQKSLSLSPGK	
AZD8 895-275	QMQLVQSGPEVKKPGTSVKVSCKASGFTFMSSAVQWVRQARGQRLEWIGWIVIGSGNTNYAQKFQERVTITRDMSTSTAYMELSSLRSEDTAVYYCAA<br>PYCSLTYCSDGFDIWGQGTMVTVSSASTKGPSVFPLAPSSKSTSGGTAALGCLVKDYFPEPVTVSWNSGALTSGVHTFPAVLQSSGLYSLSSVVTVPSSSL<br>GTQTYICNVNHKPSNTKVDKRVEPKSCDKTHTCPPCPAPEFEGGPSVFLFPPKPKDTLYITREPEVTCVVVDVSHEDPEVKFNWYVDGVEVHNAKTKPRE<br>EQYNSTYRVVSVLTVLHQDWLNGKEYKCKVSNKALPASIEKTISKAKGQPREPQVYTLPPSREEMTKNQVSLTCLVKGFYPSDIAVEWESNGQPENNYK<br>TTPPVLDSDGSFFLYSKLTVDKSRWQQGNVFSCSVMHEALHNHYTQKSLSLSPGK	
AZD8 895-449	QMQLVQSGPEVKKPGTSVKVSCKASGFTFMSSAVQWVRQARGQRLEWIGWIVIGSGNTNYAQKFQERVTITRDMSTSTAYMELSSLRSEDTAVYYCAA<br>PYCSLTNCSDGFDIWGQGTMVTVSSASTKGPSVFPLAPSSKSTSGGTAALGCLVKDYFPEPVTVSWNSGALTSGVHTFPAVLQSSGLYSLSSVVTVPSSSL<br>GTQTYICNVNHKPSNTKVDKRVEPKSCDKTHTCPPCPAPEFEGGPSVFLFPPKPKDTLYITREPEVTCVVVDVSHEDPEVKFNWYVDGVEVHNAKTKPRE<br>EQYNSTYRVVSVLTVLHQDWLNGKEYKCKVSNKALPASIEKTISKAKGQPREPQVYTLPPSREEMTKNQVSLTCLVKGFYPSDIAVEWESNGQPENNYK<br>TTPPVLDSDGSFFLYSKLTVDKSRWQQGNVFSCSVMHEALHNHYTQKSLSLSPGK	
AZD8 895-450	QMQLVQSGPEVKKPGTSVKVSCKASGFTFMSSAVQWVRQARGQRLEWIGWIVIGSGNTNYAQKFQERVTITRDMSTSTAYMELSSLRSEDTAVYYCAA<br>NYCSSTICSDGMDIWGQGTMVTVSSASTKGPSVFPLAPSSKSTSGGTAALGCLVKDYFPEPVTVSWNSGALTSGVHTFPAVLQSSGLYSLSSVVTVPSSSL<br>GTQTYICNVNHKPSNTKVDKRVEPKSCDKTHTCPPCPAPEFEGGPSVFLFPPKPKDTLYITREPEVTCVVVDVSHEDPEVKFNWYVDGVEVHNAKTKPRE<br>EQYNSTYRVVSVLTVLHQDWLNGKEYKCKVSNKALPASIEKTISKAKGQPREPQVYTLPPSREEMTKNQVSLTCLVKGFYPSDIAVEWESNGQPENNYK<br>TTPPVLDSDGSFFLYSKLTVDKSRWQQGNVFSCSVMHEALHNHYTQKSLSLSPGK	
Sotrovi mab	QVQLVQSGAEVKKPGASVKVSCKASGYPFTSYGISWVRQAPGQGLEWMGWISTYQGNTNYAQKFQGRVTMTTDTSTTTGYMELRRLRSDDTAVYYCA<br>RDYTRGAWFGESLIGGFDNWGQGTLVTVSSASTKGPSVFPLAPSSKSTSGGTAALGCLVKDYFPEPVTVSWNSGALTSGVHTFPAVLQSSGLYSLSSVVT<br>VPSSSLGTQTYICNVNHKPSNTKVDKKVEPKSCDKTHTCPPCPAPELLGGPSVFLFPPKPKDTLMISRTPEVTCVVVDVSHEDPEVKFNWYVDGVEVHNA<br>KTKPREEQYNSTYRVVSVLTVLHQDWLNGKEYKCKVSNKALPAPIEKTISKAKGQPREPQVYTLPPSRDELTKNQVSLTCLVKGFYPSDIAVEWESNGQP<br>ENNYKTTPPVLDSDGSFFLYSKLTVDKSRWQQGNVFSCSVLHEALHSHYTQKSLSLSPGK	10.1080/135 43784.2022. 2030310

Sotrovimab-203720	QVQLVQSGAEVKKPGASVKVSCKASGYPFTSYGISWVRQAPGQGLEWMGWISTYQGNTNYAQKFQGRVTMTTDTSTTTGYMELRRLRSDDTAVYYCA<br>RDRTRGYFGLASLIGGLDNWGQGTLVTVSSASTKGPSVFPLAPSSKSTSGGTAALGCLVKDYFPEPVTVSWNSGALTSGVHTFPAVLQSSGLYSLSSVVT<br>VPSSSLGTQTYICNVNHKPSNTKVDKKVEPKSCDKTHTCPPCPAPELLGGPSVFLFPPKPKDTLMISRTPEVTCVVVDVSHEDPEVKFNWYVDGVEVHNA<br>KTKPREEQYNSTYRVVSVLTVLHQDWLNGKEYKCKVSNKALPAPIEKTISKAKGQPREPQVYTLPPSRDELTKNQVSLTCLVKGFYPSDIAVEWESNGQP<br>ENNYKTTPPVLDSDGSFFLYSKLTVDKSRWQQGNVFSCSVLHEALHSHYTQKSLSLSPGK	
Sotrovimab-380606	QVQLVQSGAEVKKPGASVKVSCKASGYPFTSYGISWVRQAPGQGLEWMGWISTYQGNTNYAQKFQGRVTMTTDTSTTTGYMELRRLRSDDTAVYYCA<br>RDLTRGYFGWESLIGGLDDWGQGTLVTVSSASTKGPSVFPLAPSSKSTSGGTAALGCLVKDYFPEPVTVSWNSGALTSGVHTFPAVLQSSGLYSLSSVVT<br>VPSSSLGTQTYICNVNHKPSNTKVDKKVEPKSCDKTHTCPPCPAPELLGGPSVFLFPPKPKDTLMISRTPEVTCVVVDVSHEDPEVKFNWYVDGVEVHNA<br>KTKPREEQYNSTYRVVSVLTVLHQDWLNGKEYKCKVSNKALPAPIEKTISKAKGQPREPQVYTLPPSRDELTKNQVSLTCLVKGFYPSDIAVEWESNGQP<br>ENNYKTTPPVLDSDGSFFLYSKLTVDKSRWQQGNVFSCSVLHEALHSHYTQKSLSLSPGK	
Sotrovimab-395830	QVQLVQSGAEVKKPGASVKVSCKASGYPFTSYGISWVRQAPGQGLEWMGWISTYQGNTNYAQKFQGRVTMTTDTSTTTGYMELRRLRSDDTAVYYCA<br>RDYTRAYFGLWSLVGGLDNWGQGTLVTVSSASTKGPSVFPLAPSSKSTSGGTAALGCLVKDYFPEPVTVSWNSGALTSGVHTFPAVLQSSGLYSLSSVVT<br>VPSSSLGTQTYICNVNHKPSNTKVDKKVEPKSCDKTHTCPPCPAPELLGGPSVFLFPPKPKDTLMISRTPEVTCVVVDVSHEDPEVKFNWYVDGVEVHNA<br>KTKPREEQYNSTYRVVSVLTVLHQDWLNGKEYKCKVSNKALPAPIEKTISKAKGQPREPQVYTLPPSRDELTKNQVSLTCLVKGFYPSDIAVEWESNGQP<br>ENNYKTTPPVLDSDGSFFLYSKLTVDKSRWQQGNVFSCSVLHEALHSHYTQKSLSLSPGK	
Sotrovimab-413456	QVQLVQSGAEVKKPGASVKVSCKASGYPFTSYGISWVRQAPGQGLEWMGWISTYQGNTNYAQKFQGRVTMTTDTSTTTGYMELRRLRSDDTAVYYCA<br>RDLTRGYFGWESLLGGIDNWGQGTLVTVSSASTKGPSVFPLAPSSKSTSGGTAALGCLVKDYFPEPVTVSWNSGALTSGVHTFPAVLQSSGLYSLSSVVT<br>VPSSSLGTQTYICNVNHKPSNTKVDKKVEPKSCDKTHTCPPCPAPELLGGPSVFLFPPKPKDTLMISRTPEVTCVVVDVSHEDPEVKFNWYVDGVEVHNA<br>KTKPREEQYNSTYRVVSVLTVLHQDWLNGKEYKCKVSNKALPAPIEKTISKAKGQPREPQVYTLPPSRDELTKNQVSLTCLVKGFYPSDIAVEWESNGQP<br>ENNYKTTPPVLDSDGSFFLYSKLTVDKSRWQQGNVFSCSVLHEALHSHYTQKSLSLSPGK	
Sotrovimab-425504	QVQLVQSGAEVKKPGASVKVSCKASGYPFTSYGISWVRQAPGQGLEWMGWISTYQGNTNYAQKFQGRVTMTTDTSTTTGYMELRRLRSDDTAVYYCA<br>RDLTRGFFGTASLLGGFDNWGQGTLVTVSSASTKGPSVFPLAPSSKSTSGGTAALGCLVKDYFPEPVTVSWNSGALTSGVHTFPAVLQSSGLYSLSSVVT<br>VPSSSLGTQTYICNVNHKPSNTKVDKKVEPKSCDKTHTCPPCPAPELLGGPSVFLFPPKPKDTLMISRTPEVTCVVVDVSHEDPEVKFNWYVDGVEVHNA<br>KTKPREEQYNSTYRVVSVLTVLHQDWLNGKEYKCKVSNKALPAPIEKTISKAKGQPREPQVYTLPPSRDELTKNQVSLTCLVKGFYPSDIAVEWESNGQP<br>ENNYKTTPPVLDSDGSFFLYSKLTVDKSRWQQGNVFSCSVLHEALHSHYTQKSLSLSPGK	
REGN 10987	QVQLVESGGGVVQPGRSLRLSCAASGFTFSNYAMYWVRQAPGKGLEWVAVISYDGSNKYYADSVKGRFTISRDNSKNTLYLQMNSLRTEDTAVYYCA<br>SGSDYGDYLLVYWGQGTLVTVSSASTKGPSVFPLAPSSKSTSGGTAALGCLVKDYFPEPVTVSWNSGALTSGVHTFPAVLQSSGLYSLSSVVTVPSSSLGT<br>QTYICNVNHKPSNTKVDKKVEPKSCDKTHTCPPCPAPELLGGPSVFLFPPKPKDTLMISRTPEVTCVVVDVSHEDPEVKFNWYVDGVEVHNAKTKPREEQ<br>YNSTYRVVSVLTVLHQDWLNGKEYKCKVSNKALPAPIEKTISKAKGQPREPQVYTLPPSRDELTKNQVSLTCLVKGFYPSDIAVEWESNGQPENNYKTTP<br>PVLDSDGSFFLYSKLTVDKSRWQQGNVFSCSVMHEALHNHYTQKSLSLSPGK	10.1126/science.abe2402
REGN 10987-18665	QVQLVESGGGVVQPGRSLRLSCAASGFTFSNYAMYWVRQAPGKGLEWVAVISYDGSNKYYADSVKGRFTISRDNSKNTLYLQMNSLRTEDTAVYYCA<br>SGFDYVLWLLYYWGQGTLVTVSSASTKGPSVFPLAPSSKSTSGGTAALGCLVKDYFPEPVTVSWNSGALTSGVHTFPAVLQSSGLYSLSSVVTVPSSSLG<br>TQTYICNVNHKPSNTKVDKKVEPKSCDKTHTCPPCPAPELLGGPSVFLFPPKPKDTLMISRTPEVTCVVVDVSHEDPEVKFNWYVDGVEVHNAKTKPREE<br>QYNSTYRVVSVLTVLHQDWLNGKEYKCKVSNKALPAPIEKTISKAKGQPREPQVYTLPPSRDELTKNQVSLTCLVKGFYPSDIAVEWESNGQPENNYKT<br>TPPVLDSDGSFFLYSKLTVDKSRWQQGNVFSCSVMHEALHNHYTQKSLSLSPGK	
REGN 10987-24622	QVQLVESGGGVVQPGRSLRLSCAASGFTFSNYAMYWVRQAPGKGLEWVAVISYDGSNKYYADSVKGRFTISRDNSKNTLYLQMNSLRTEDTAVYYCA<br>SGFDYVYLLLAYWGQGTLVTVSSASTKGPSVFPLAPSSKSTSGGTAALGCLVKDYFPEPVTVSWNSGALTSGVHTFPAVLQSSGLYSLSSVVTVPSSSLGT<br>QTYICNVNHKPSNTKVDKKVEPKSCDKTHTCPPCPAPELLGGPSVFLFPPKPKDTLMISRTPEVTCVVVDVSHEDPEVKFNWYVDGVEVHNAKTKPREEQ<br>YNSTYRVVSVLTVLHQDWLNGKEYKCKVSNKALPAPIEKTISKAKGQPREPQVYTLPPSRDELTKNQVSLTCLVKGFYPSDIAVEWESNGQPENNYKTTP<br>PVLDSDGSFFLYSKLTVDKSRWQQGNVFSCSVMHEALHNHYTQKSLSLSPGK	

REGN 10987-25075	QVQLVESGGGVVQPGRSLRLSCAASGFTFSNYAMYWVRQAPGKGLEWVAVISYDGSNKYYADSVKGRFTISRDNSKNTLYLQMNSLRTEDTAVYYCA<br>SGFDYDAYLLYYWGQGTLVTVSSASTKGPSVFPLAPSSKSTSGGTAALGCLVKDYFPEPVTVSWNSGALTSGVHTFPAVLQSSGLYSLSSVVTVPSSSLGT<br>QTYICNVNHKPSNTKVDKKVEPKSCDKTHTCPPCPAPELLGGPSVFLFPPKPKDTLMISRTPEVTCVVVDVSHEDPEVKFNWYVDGVEVHNAKTKPREEQ<br>YNSTYRVVSVLTVLHQDWLNGKEYKCKVSNKALPAPIEKTISKAKGQPREPQVYTLPPSRDELTKNQVSLTCLVKGFYPSDIAVEWESNGQPENNYKTTP<br>PVLDSDGSFFLYSKLTVDKSRWQQGNVFSCSVMHEALHNHYTQKSLSLSPGK	
REGN 10987-28158	QVQLVESGGGVVQPGRSLRLSCAASGFTFSNYAMYWVRQAPGKGLEWVAVISYDGSNKYYADSVKGRFTISRDNSKNTLYLQMNSLRTEDTAVYYCA<br>NGSDYFLYLLYYWGQGTLVTVSSASTKGPSVFPLAPSSKSTSGGTAALGCLVKDYFPEPVTVSWNSGALTSGVHTFPAVLQSSGLYSLSSVVTVPSSSLGT<br>QTYICNVNHKPSNTKVDKKVEPKSCDKTHTCPPCPAPELLGGPSVFLFPPKPKDTLMISRTPEVTCVVVDVSHEDPEVKFNWYVDGVEVHNAKTKPREEQ<br>YNSTYRVVSVLTVLHQDWLNGKEYKCKVSNKALPAPIEKTISKAKGQPREPQVYTLPPSRDELTKNQVSLTCLVKGFYPSDIAVEWESNGQPENNYKTTP<br>PVLDSDGSFFLYSKLTVDKSRWQQGNVFSCSVMHEALHNHYTQKSLSLSPGK	
REGN 10987-6516	QVQLVESGGGVVQPGRSLRLSCAASGFTFSNYAMYWVRQAPGKGLEWVAVISYDGSNKYYADSVKGRFTISRDNSKNTLYLQMNSLRTEDTAVYYCA<br>WGSDYVLLLLYYWGQGTLVTVSSASTKGPSVFPLAPSSKSTSGGTAALGCLVKDYFPEPVTVSWNSGALTSGVHTFPAVLQSSGLYSLSSVVTVPSSSLG<br>TQTYICNVNHKPSNTKVDKKVEPKSCDKTHTCPPCPAPELLGGPSVFLFPPKPKDTLMISRTPEVTCVVVDVSHEDPEVKFNWYVDGVEVHNAKTKPREE<br>QYNSTYRVVSVLTVLHQDWLNGKEYKCKVSNKALPAPIEKTISKAKGQPREPQVYTLPPSRDELTKNQVSLTCLVKGFYPSDIAVEWESNGQPENNYKT<br>TPPVLDSDGSFFLYSKLTVDKSRWQQGNVFSCSVMHEALHNHYTQKSLSLSPGK	
MW05	EVQLVQSGAEVKKPGSSVKVSCKASGGTFSSYAISWVRQAPGQGLEWMGRIIPIFGSSNYAQKFQGRVTITADESTSTAYMELSSLRSEDTAVYYCAESPL<br>GGGSGYSVSWFDPWGQGTLVTVSSASTKGPSVFPLAPSSKSTSGGTAALGCLVKDYFPEPVTVSWNSGALTSGVHTFPAVLQSSGLYSLSSVVTVPSSSLG<br>TQTYICNVNHKPSNTKVDKRVEPKSCDKTHTCPPCPAPEFEGGPSVFLFPPKPKDTLYITREPEVTCVVVDVSHEDPEVKFNWYVDGVEVHNAKTKPREE<br>QYNSTYRVVSVLTVLHQDWLNGKEYKCKVSNKALPASIEKTISKAKGQPREPQVYTLPPSREEMTKNQVSLTCLVKGFYPSDIAVEWESNGQPENNYKT<br>TPPVLDSDGSFFLYSKLTVDKSRWQQGNVFSCSVMHEALHNHYTQKSLSLSPGK	10.1038/s41467-020-19568-1
MW05-110198	EVQLVQSGAEVKKPGSSVKVSCKASGGTFSSYAISWVRQAPGQGLEWMGRIIPIFGSSNYAQKFQGRVTITADESTSTAYMELSSLRSEDTAVYYCASSPC<br>GGISWYWVSWFDPWGQGTLVTVSSASTKGPSVFPLAPSSKSTSGGTAALGCLVKDYFPEPVTVSWNSGALTSGVHTFPAVLQSSGLYSLSSVVTVPSSSL<br>GTQTYICNVNHKPSNTKVDKRVEPKSCDKTHTCPPCPAPEFEGGPSVFLFPPKPKDTLYITREPEVTCVVVDVSHEDPEVKFNWYVDGVEVHNAKTKPRE<br>EQYNSTYRVVSVLTVLHQDWLNGKEYKCKVSNKALPASIEKTISKAKGQPREPQVYTLPPSREEMTKNQVSLTCLVKGFYPSDIAVEWESNGQPENNYK<br>TTPPVLDSDGSFFLYSKLTVDKSRWQQGNVFSCSVMHEALHNHYTQKSLSLSPGK	
MW05-112489	EVQLVQSGAEVKKPGSSVKVSCKASGGTFSSYAISWVRQAPGQGLEWMGRIIPIFGSSNYAQKFQGRVTITADESTSTAYMELSSLRSEDTAVYYCAHSPS<br>GGFRCYSVSWFDPWGQGTLVTVSSASTKGPSVFPLAPSSKSTSGGTAALGCLVKDYFPEPVTVSWNSGALTSGVHTFPAVLQSSGLYSLSSVVTVPSSSLG<br>TQTYICNVNHKPSNTKVDKRVEPKSCDKTHTCPPCPAPEFEGGPSVFLFPPKPKDTLYITREPEVTCVVVDVSHEDPEVKFNWYVDGVEVHNAKTKPREE<br>QYNSTYRVVSVLTVLHQDWLNGKEYKCKVSNKALPASIEKTISKAKGQPREPQVYTLPPSREEMTKNQVSLTCLVKGFYPSDIAVEWESNGQPENNYKT<br>TPPVLDSDGSFFLYSKLTVDKSRWQQGNVFSCSVMHEALHNHYTQKSLSLSPGK	
MW05-126313	EVQLVQSGAEVKKPGSSVKVSCKASGGTFSSYAISWVRQAPGQGLEWMGRIIPIFGSSNYAQKFQGRVTITADESTSTAYMELSSLRSEDTAVYYCATSPC<br>GGGSWYYVRWFDPWGQGTLVTVSSASTKGPSVFPLAPSSKSTSGGTAALGCLVKDYFPEPVTVSWNSGALTSGVHTFPAVLQSSGLYSLSSVVTVPSSSL<br>GTQTYICNVNHKPSNTKVDKRVEPKSCDKTHTCPPCPAPEFEGGPSVFLFPPKPKDTLYITREPEVTCVVVDVSHEDPEVKFNWYVDGVEVHNAKTKPRE<br>EQYNSTYRVVSVLTVLHQDWLNGKEYKCKVSNKALPASIEKTISKAKGQPREPQVYTLPPSREEMTKNQVSLTCLVKGFYPSDIAVEWESNGQPENNYK<br>TTPPVLDSDGSFFLYSKLTVDKSRWQQGNVFSCSVMHEALHNHYTQKSLSLSPGK	
MW05-40147	EVQLVQSGAEVKKPGSSVKVSCKASGGTFSSYAISWVRQAPGQGLEWMGRIIPIFGSSNYAQKFQGRVTITADESTSTAYMELSSLRSEDTAVYYCASSPS<br>GGLRCYSVSWFDPWGQGTLVTVSSASTKGPSVFPLAPSSKSTSGGTAALGCLVKDYFPEPVTVSWNSGALTSGVHTFPAVLQSSGLYSLSSVVTVPSSSLG<br>TQTYICNVNHKPSNTKVDKRVEPKSCDKTHTCPPCPAPEFEGGPSVFLFPPKPKDTLYITREPEVTCVVVDVSHEDPEVKFNWYVDGVEVHNAKTKPREE<br>QYNSTYRVVSVLTVLHQDWLNGKEYKCKVSNKALPASIEKTISKAKGQPREPQVYTLPPSREEMTKNQVSLTCLVKGFYPSDIAVEWESNGQPENNYKT<br>TPPVLDSDGSFFLYSKLTVDKSRWQQGNVFSCSVMHEALHNHYTQKSLSLSPGK	

MW05-68299	EVQLVQSGAEVKKPGSSVKVSCKASGGTFSSYAISWVRQAPGQGLEWMGRIIPIFGSSNYAQKFQGRVTITADESTSTAYMELSSLRSEDTAVYYCAHSPS<br>GGLSGFNVSWFDPWGQGTLVTVSSASTKGPSVFPLAPSSKSTSGGTAALGCLVKDYFPEPVTVSWNSGALTSGVHTFPAVLQSSGLYSLSSVVTVPSSSLG<br>TQTYICNVNHKPSNTKVDKRVEPKSCDKTHTCPPCPAPEFEGGPSVFLFPPKPKDTLYITREPEVTCVVVDVSHEDPEVKFNWYVDGVEVHNAKTKPREE<br>QYNSTYRVVSVLTVLHQDWLNGKEYKCKVSNKALPASIEKTISKAKGQPREPQVYTLPPSREEMTKNQVSLTCLVKGFYPSDIAVEWESNGQPENNYKT<br>TPPVLDSDGSFFLYSKLTVDKSRWQQGNVFSCSVMHEALHNHYTQKSLSLSPGK	
REGN 10933	QVQLVESGGGLVKPGGSLRLSCAASGFTFSDYYMSWIRQAPGKGLEWVSYITYSGSTIYYADSVKGRFTISRDNAKSSLYLQMNSLRAEDTAVYYCARD<br>RGTTMVPFDYWGQGTLVTVSSASTKGPSVFPLAPSSKSTSGGTAALGCLVKDYFPEPVTVSWNSGALTSGVHTFPAVLQSSGLYSLSSVVTVPSSSLGTQT<br>YICNVNHKPSNTKVDKKVEPKSCDKTHTCPPCPAPELLGGPSVFLFPPKPKDTLMISRTPEVTCVVVDVSHEDPEVKFNWYVDGVEVHNAKTKPREEQY<br>NSTYRVVSVLTVLHQDWLNGKEYKCKVSNKALPAPIEKTISKAKGQPREPQVYTLPPSRDELTKNQVSLTCLVKGFYPSDIAVEWESNGQPENNYKTTPP<br>VLDSDGSFFLYSKLTVDKSRWQQGNVFSCSVMHEALHNHYTQKSLSLSPGK	10.1126/science.abe2402
REGN 10933-13041	QVQLVESGGGLVKPGGSLRLSCAASGFTFSDYYMSWIRQAPGKGLEWVSYITYSGSTIYYADSVKGRFTISRDNAKSSLYLQMNSLRAEDTAVYYCARK<br>HGYTMVLYDYWGQGTLVTVSSASTKGPSVFPLAPSSKSTSGGTAALGCLVKDYFPEPVTVSWNSGALTSGVHTFPAVLQSSGLYSLSSVVTVPSSSLGTQ<br>TYICNVNHKPSNTKVDKKVEPKSCDKTHTCPPCPAPELLGGPSVFLFPPKPKDTLMISRTPEVTCVVVDVSHEDPEVKFNWYVDGVEVHNAKTKPREEQY<br>NSTYRVVSVLTVLHQDWLNGKEYKCKVSNKALPAPIEKTISKAKGQPREPQVYTLPPSRDELTKNQVSLTCLVKGFYPSDIAVEWESNGQPENNYKTTPP<br>VLDSDGSFFLYSKLTVDKSRWQQGNVFSCSVMHEALHNHYTQKSLSLSPGK	
REGN 10933-15539	QVQLVESGGGLVKPGGSLRLSCAASGFTFSDYYMSWIRQAPGKGLEWVSYITYSGSTIYYADSVKGRFTISRDNAKSSLYLQMNSLRAEDTAVYYCARK<br>RGYTMDMIDYWGQGTLVTVSSASTKGPSVFPLAPSSKSTSGGTAALGCLVKDYFPEPVTVSWNSGALTSGVHTFPAVLQSSGLYSLSSVVTVPSSSLGTQ<br>TYICNVNHKPSNTKVDKKVEPKSCDKTHTCPPCPAPELLGGPSVFLFPPKPKDTLMISRTPEVTCVVVDVSHEDPEVKFNWYVDGVEVHNAKTKPREEQY<br>NSTYRVVSVLTVLHQDWLNGKEYKCKVSNKALPAPIEKTISKAKGQPREPQVYTLPPSRDELTKNQVSLTCLVKGFYPSDIAVEWESNGQPENNYKTTPP<br>VLDSDGSFFLYSKLTVDKSRWQQGNVFSCSVMHEALHNHYTQKSLSLSPGK	
REGN 10933-15647	QVQLVESGGGLVKPGGSLRLSCAASGFTFSDYYMSWIRQAPGKGLEWVSYITYSGSTIYYADSVKGRFTISRDNAKSSLYLQMNSLRAEDTAVYYCARK<br>YGYTMVPFDYWGQGTLVTVSSASTKGPSVFPLAPSSKSTSGGTAALGCLVKDYFPEPVTVSWNSGALTSGVHTFPAVLQSSGLYSLSSVVTVPSSSLGTQ<br>TYICNVNHKPSNTKVDKKVEPKSCDKTHTCPPCPAPELLGGPSVFLFPPKPKDTLMISRTPEVTCVVVDVSHEDPEVKFNWYVDGVEVHNAKTKPREEQY<br>NSTYRVVSVLTVLHQDWLNGKEYKCKVSNKALPAPIEKTISKAKGQPREPQVYTLPPSRDELTKNQVSLTCLVKGFYPSDIAVEWESNGQPENNYKTTPP<br>VLDSDGSFFLYSKLTVDKSRWQQGNVFSCSVMHEALHNHYTQKSLSLSPGK	
REGN 10933-16792	QVQLVESGGGLVKPGGSLRLSCAASGFTFSDYYMSWIRQAPGKGLEWVSYITYSGSTIYYADSVKGRFTISRDNAKSSLYLQMNSLRAEDTAVYYCARK<br>WGYTMVYPDYWGQGTLVTVSSASTKGPSVFPLAPSSKSTSGGTAALGCLVKDYFPEPVTVSWNSGALTSGVHTFPAVLQSSGLYSLSSVVTVPSSSLGTQ<br>TYICNVNHKPSNTKVDKKVEPKSCDKTHTCPPCPAPELLGGPSVFLFPPKPKDTLMISRTPEVTCVVVDVSHEDPEVKFNWYVDGVEVHNAKTKPREEQY<br>NSTYRVVSVLTVLHQDWLNGKEYKCKVSNKALPAPIEKTISKAKGQPREPQVYTLPPSRDELTKNQVSLTCLVKGFYPSDIAVEWESNGQPENNYKTTPP<br>VLDSDGSFFLYSKLTVDKSRWQQGNVFSCSVMHEALHNHYTQKSLSLSPGK	
REGN 10933-8224	QVQLVESGGGLVKPGGSLRLSCAASGFTFSDYYMSWIRQAPGKGLEWVSYITYSGSTIYYADSVKGRFTISRDNAKSSLYLQMNSLRAEDTAVYYCARK<br>AGYTMVKFDYWGQGTLVTVSSASTKGPSVFPLAPSSKSTSGGTAALGCLVKDYFPEPVTVSWNSGALTSGVHTFPAVLQSSGLYSLSSVVTVPSSSLGTQ<br>TYICNVNHKPSNTKVDKKVEPKSCDKTHTCPPCPAPELLGGPSVFLFPPKPKDTLMISRTPEVTCVVVDVSHEDPEVKFNWYVDGVEVHNAKTKPREEQY<br>NSTYRVVSVLTVLHQDWLNGKEYKCKVSNKALPAPIEKTISKAKGQPREPQVYTLPPSRDELTKNQVSLTCLVKGFYPSDIAVEWESNGQPENNYKTTPP<br>VLDSDGSFFLYSKLTVDKSRWQQGNVFSCSVMHEALHNHYTQKSLSLSPGK	

Supplementary Table 3. RMSD in the structure of seed and its optimized antibodies.

NMAb name	Seed | Optimized	Neutralizing activity	H3 CDR stabilized	RMSD after refinement	Number of aligned atoms after refinement	Number of refinement cycles	RMSD before refinement	Number of aligned atoms before refinement	Raw alignment score	Number of residues aligned
AZD8895	Seed	√	√							
AZD8895-25	Optimized	√		2.2262	3442	4	2.3214	3494	2395.0	453
AZD8895-275	Optimized	√		0.7887	3436	5	0.8569	3494	2394.0	453
AZD8895-449	Optimized	√		0.6398	3425	4	0.7195	3494	2397.0	453
AZD8895-450	Optimized	√		0.5416	3237	5	0.6997	3489	2385.0	453
Mean±Std				1.0787±0.6885						
Sotrovimab	Seed	√	x							
Sotrovimab-203720	Optimized			1.7876	3439	5	1.9173	3503	2394.0	456
Sotrovimab-380606	Optimized			0.4633	2749	5	0.9853	3505	2396.0	457
Sotrovimab-395830	Optimized			6.9516	3422	5	7.2817	3509	2398.0	456
Sotrovimab-413456	Optimized			0.3479	2741	5	0.9948	3502	2399.0	457
Sotrovimab-425504	Optimized			7.1564	3458	4	7.3288	3503	2399.0	457
Mean±Std				3.3413±3.4368						
REGN10987	Seed	√	x							
REGN10987-18665	Optimized			1.4704	3031	5	2.0682	3464	2364.0	450
REGN10987-24622	Optimized			0.9575	2863	5	1.6636	3463	2363.0	450
REGN10987-25075	Optimized			0.8053	3116	5	1.0861	3466	2373.0	450
REGN10987-28158	Optimized			1.7384	3331	5	1.9628	3467	2372.0	450
REGN10987-6516	Optimized			1.9567	3057	5	2.6927	3463	2360.0	450
Mean±Std				1.3857±0.4944						
MW05	Seed	√	x							
MW05-110198	Optimized			1.7743	3237	5	2.0819	3480	2385.0	455

MW05-112489	Optimized			0.6364	3290	5	0.7749	3481	2386.0	455
MW05-126313	Optimized			0.9416	3243	5	1.4031	3479	2390.0	455
MW05-40147	Optimized			0.6976	3334	5	0.8565	3480	2385.0	455
MW05-68299	Optimized			0.3992	2810	5	0.8088	3480	2392.0	455
Mean±Std				0.8898±0.5308						
REGN10933	Seed	√	x							
REGN10933-13041	Optimized			0.4857	3049	5	0.7724	3461	2366.0	450
REGN10933-15539	Optimized			0.2072	3135	5	0.4896	3459	2362.0	450
REGN10933-15647	Optimized			0.1153	3164	5	0.3812	3463	2377.0	450
REGN10933-16792	Optimized			0.2114	3213	5	0.6179	3456	2356.0	450
REGN10933-8224	Optimized			0.1876	3061	5	0.4345	3461	2370.0	450
Mean±Std				0.2414±0.1419						

Supplementary Table 4. AZD8895_mt_97to116_clean_ddg_results

mutation	ddg	wt_score	mut_score	status	error	position	wild_type	mutant	position_wt		mutation
P3N	-55.7979	95.68001	39.88214	success		3	P	N	99	P99N	P99N
S6Y	5.396146	95.68001	101.0762	success		6	S	Y	102	S102Y	S102Y
S7L	387.3838	95.68001	483.0638	success		7	S	L	103	S103L	S103L
S9C	0.034184	95.68001	95.71419	success		9	S	C	105	S105C	S105C
S9Y	-0.64744	95.68001	95.03257	success		9	S	Y	105	S105Y	S105Y
S9C	0.034184	95.68001	95.71419	success		9	S	C	105	S105C	S105C
S9N	-9.8234	95.68001	85.8566	success		9	S	N	105	S105N	S105N
S9I	39.73354	95.68001	135.4135	success		9	S	I	105	S105I	S105I
N11S	-0.11958	95.68001	95.56043	success		11	N	S	107	N107S	N107S
F14M	-37.674	95.68001	58.00603	success		14	F	M	110	F110M	F110M

Supplementary Table 5. AZD8895_wt_97to116_clean_ddg_results

mutation	ddg	wt_score	mut_score	status	error	position	wild_type	mutant	position_wt		mutation
P3N	3.156932	-17.0094	-13.8524	success		3	P	N	99	P3N	3.156932
S6Y	8.366587	-17.0094	-8.64278	success		6	S	Y	102	S6Y	8.366587
S7L	6.343628	-17.0094	-10.6657	success		7	S	L	103	S7L	6.343628
S9C	4.810082	-17.0094	-12.1993	success		9	S	C	105	S9C	4.810082
S9Y	0.129655	-17.0094	-16.8797	success		9	S	Y	105	S9Y	0.129655
S9C	4.810082	-17.0094	-12.1993	success		9	S	C	105	S9C	4.810082
S9N	9.662113	-17.0094	-7.34725	success		9	S	N	105	S9N	9.662113
S9I	7.288091	-17.0094	-9.72127	success		9	S	I	105	S9I	7.288091
N11S	7.546301	-17.0094	-9.46306	success		11	N	S	107	N11S	7.546301
F14M	-0.06492	-17.0094	-17.0743	success		14	F	M	110	F14M	-0.06492

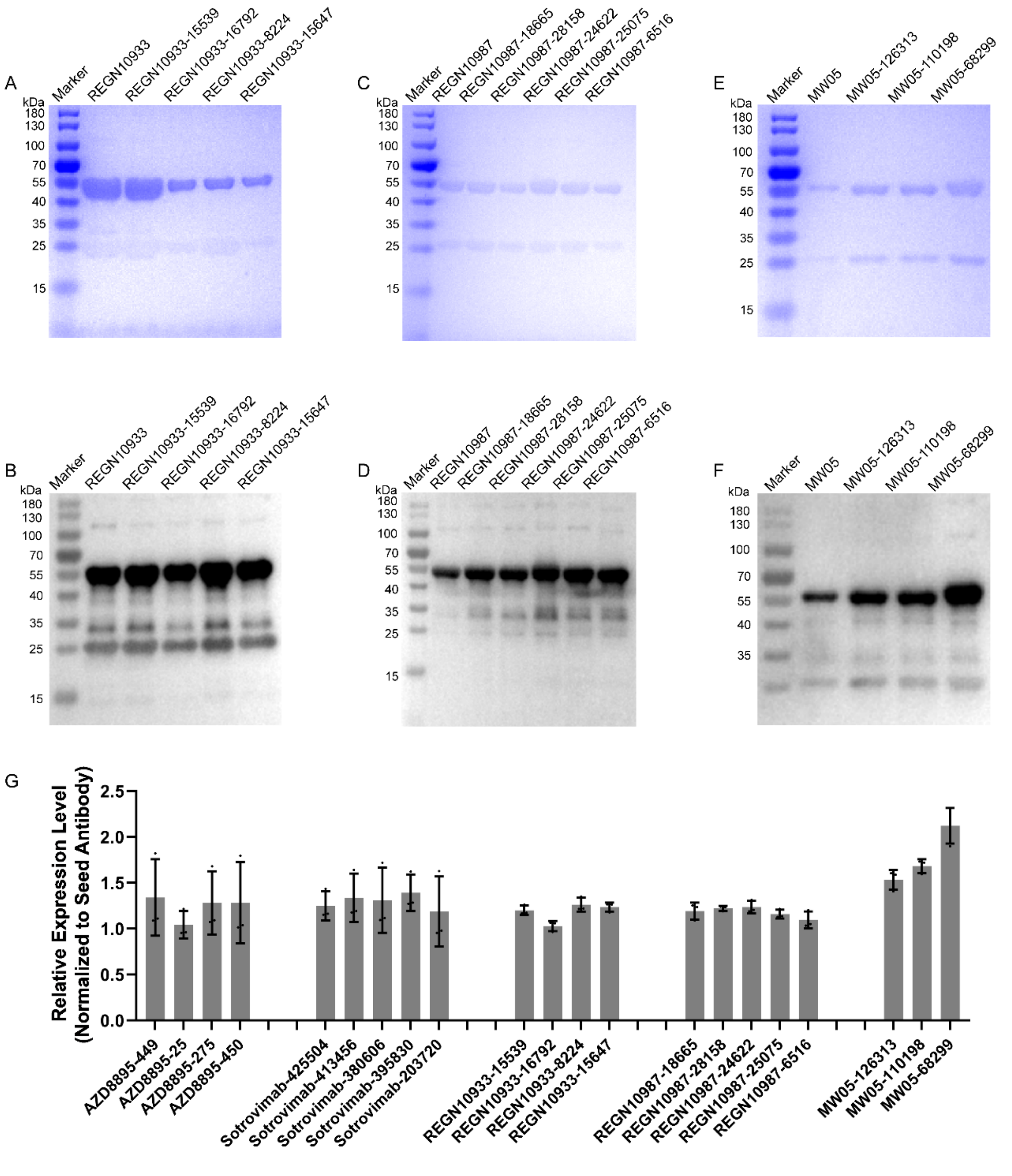

Figure S1. Expression of NMAbs. (A) SDS-PAGE analyses of the seed antibody REGN10933 and other four optimized NMAbs. The molecular weight marker (in kDa) is indicated on the left. (B) Western blot analyses of the seed antibody REGN10933 and other four optimized NMAbs. The molecular weight marker (in kDa) is indicated on the right. (C) SDS-PAGE analyses of the seed antibody REGN10987 and other five optimized NMAbs. (D) Western blot analyses of the seed antibody REGN10987 and other five optimized NMAbs. (E) SDS-PAGE analyses of the seed antibody MW05 and other three optimized NMAbs. (F) Western blot analyses of the seed antibody MW05 and other three optimized NMAbs. (G) Quantification of western blot analysis for antibody expression. The expression level of each optimized antibody was quantified and normalized to the intensity of its corresponding seed antibody from the same blot. Data are presented as mean ± SD.

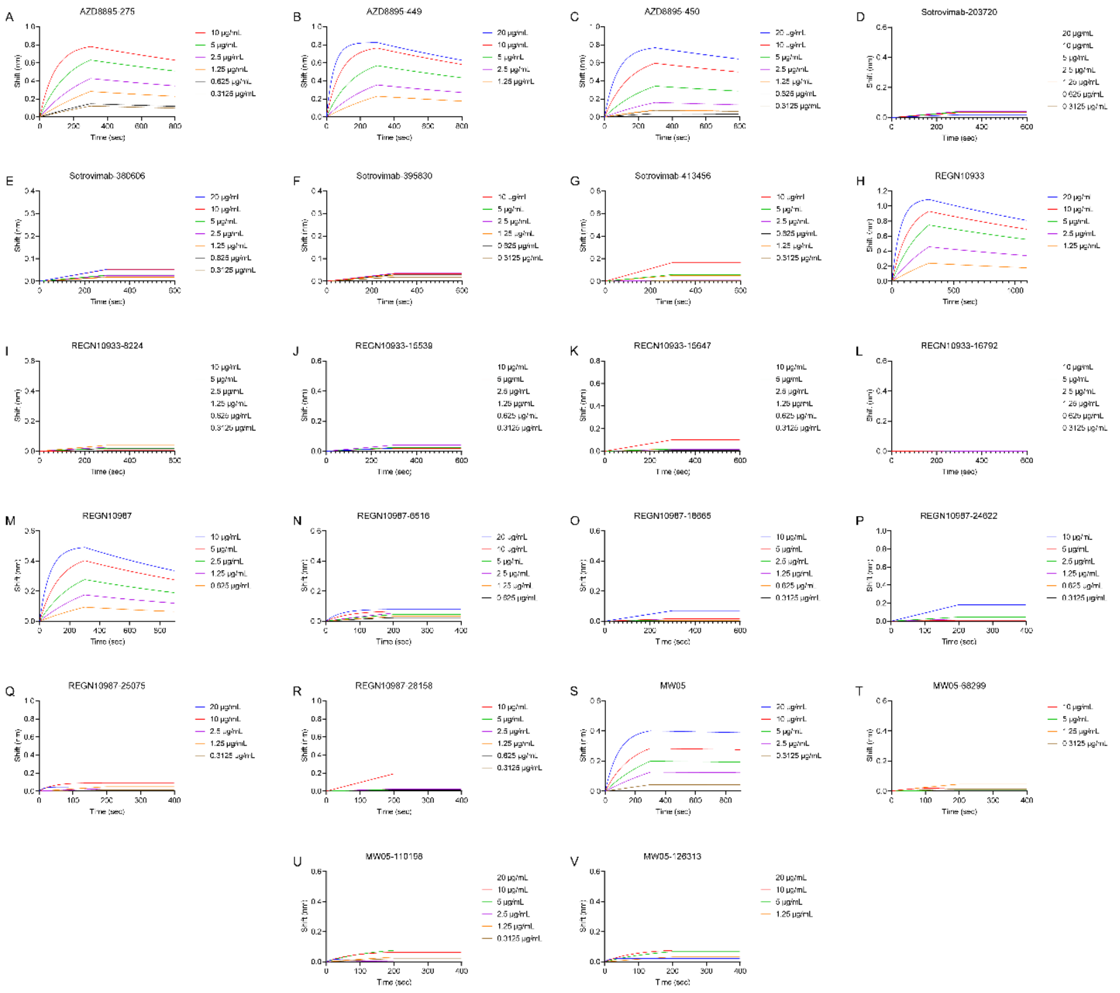

Figure S2. Binding kinetics between NMAbs and SARS-CoV-2 spike protein measured by BLI. Sensorgrams are grouped by seed antibody. (A-C) Binding kinetics of the optimized variants of AZD8895. (D-G) Binding kinetics of the optimized variants of Sotrovimab. (H-L) Binding kinetics of REGN10933 and its optimized variants. (M-R) Binding kinetics of REGN10987 and its optimized variants. (S-V) Binding kinetics of MW05 and its optimized variants. Individual antibody names are labeled above each sensorgram.

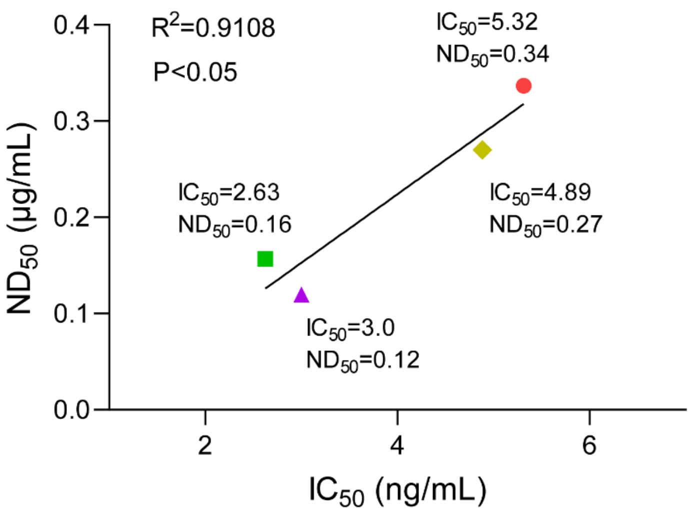

Figure S3. Analysis of the correlation between IC_{50} and ND_{50} values for antibodies AZD8895-275, AZD8895-25, AZD8895-449, and AZD8895. R^2 indicates the correlation between IC_{50} and ND_{50}.
